# Role of Pirh2 in Mediating the Regulation of p53 and c-Myc

**DOI:** 10.1371/journal.pgen.1002360

**Published:** 2011-11-17

**Authors:** Anne Hakem, Miyuki Bohgaki, Bénédicte Lemmers, Elisabeth Tai, Leonardo Salmena, Elzbieta Matysiak-Zablocki, Yong-Sam Jung, Jana Karaskova, Lilia Kaustov, Shili Duan, Jason Madore, Paul Boutros, Yi Sheng, Marta Chesi, P. Leif Bergsagel, Bayardo Perez-Ordonez, Anne-Marie Mes-Masson, Linda Penn, Jeremy Squire, Xinbin Chen, Igor Jurisica, Cheryl Arrowsmith, Otto Sanchez, Samuel Benchimol, Razqallah Hakem

**Affiliations:** 1 Ontario Cancer Institute, University Health Network and Department of Medical Biophysics, University of Toronto, Toronto, Canada; 2 Institut de Génétique Moléculaire de Montpellier CNRS-UMR 5535, Montpellier, France; 3 Department of Biology, York University, Toronto, Canada; 4 Cancer Genetics Program, Beth Israel Deaconess Cancer Center, Department of Medicine, Harvard Medical School, Boston, Massachusetts, United States of America; 5 University of California Davis, Davis, California, United States of America; 6 Institut du Cancer de Montréal, Montréal, Canada; 7 Comprehensive Cancer Center, Mayo Clinic Arizona, Scottsdale, Arizona, United States of America; 8 University Health Network and Department of Pathology, University of Toronto, Toronto, Canada; 9 Queen's University, Kingston, Canada; 10 University of Ontario Institute of Technology, Oshawa, Canada; University of Washington, United States of America

## Abstract

Ubiquitylation is fundamental for the regulation of the stability and function of p53 and c-Myc. The E3 ligase Pirh2 has been reported to polyubiquitylate p53 and to mediate its proteasomal degradation. Here, using Pirh2 deficient mice, we report that Pirh2 is important for the *in vivo* regulation of p53 stability in response to DNA damage. We also demonstrate that c-Myc is a novel interacting protein for Pirh2 and that Pirh2 mediates its polyubiquitylation and proteolysis. *Pirh2* mutant mice display elevated levels of c-Myc and are predisposed for plasma cell hyperplasia and tumorigenesis. Consistent with the role p53 plays in suppressing c-Myc-induced oncogenesis, its deficiency exacerbates tumorigenesis of *Pirh2^−/−^* mice. We also report that low expression of human *PIRH2* in lung, ovarian, and breast cancers correlates with decreased patients' survival. Collectively, our data reveal the *in vivo* roles of Pirh2 in the regulation of p53 and c-Myc stability and support its role as a tumor suppressor.

## Author Summary

Tumor suppressors and oncogenes play critical roles in cancer development. The tumor suppressor p53 and the oncogene c-MYC are among the most frequently deregulated genes in human cancer, and their ubiquitylation mediated by several E3 ligases is critical for their turnover and their functions. P53 has been shown to be ubiquitylated by Pirh2; however, the physiological significance of this modification of p53 remains unknown. In this study we have generated mice deficient for Pirh2 and have observed that loss of Pirh2 results in a higher level of p53 and cell death, especially in response to radiation. Remarkably, we also identified that Pirh2 interacts with c-Myc and mediates its polyubiquitylation and degradation. c-Myc accumulates in the absence of Pirh2 and this accumulation is accompanied by increased tumorigenesis of Pirh2-deficient mice. We also report that dual deficiency of Pirh2 and p53 synergizes cancer development. Examination of the expression level of PIRH2 in human cancers indicated that its lower expression level associates with poor survival of patients with lung, ovarian, or breast cancers. Collectively, these data indentify Pirh2 as a novel tumor suppressor involved in the regulation of both p53 and c-Myc.

## Introduction

Protein ubiquitylation is essential for a broad spectrum of cellular processes including nuclear export, endocytosis, transcriptional regulation, DNA damage repair and proteasomal degradation [1]. Impairment of the ubiquitylation process has been associated with a variety of human diseases including autoimmunity, immunodeficiency, inflammation and cancer [2], [3].

One well established role for ubiquitylation in cancer is its function in regulating the stability and function of the tumor suppressor p53 and the oncogene c-MYC, two proteins with major roles in human malignancies [2]–[4]. Monoubiquitylation of p53 serves to signal its nuclear export as well as its mitochondrial translocation, while p53 polyubiquitylation targets it for proteasomal degradation. Remarkably, p53 is targeted for ubiquitylation by several ubiquitin E3 ligases including Pirh2 (Rchy1), Mdm2/Hdm2, Cop1, E6/E6AP, ARF-BP1, Synoviolin and by atypical E3 ligases including E4F1 [5].

Similar to p53, ubiquitylation is critical for the regulation of the function and stability of c-MYC, an oncoprotein frequently overexpressed in various human cancers including breast and ovarian cancer [6], [7]. The ubiquitin ligases FBW7, SKP2 and ARF-BP1/HectH9 have been shown to mediate c-MYC ubiquitylation. While c-MYC polyubiquitylation by FBW7 leads to its proteasomal degradation, its polyubiquitylation by ARF-BP1 increases its transcriptional activity. c-MYC ubiquitylation by SKP2 has been shown to mediate both its transactivation activity and proteasomal degradation [2], [6]. Interestingly, some of the E3 ligases important for the regulation of c-Myc also regulate p53 function. SKP2 negatively regulates p53 by suppressing its acetylation by p300 while ARF-BP1 suppresses p53 through its polyubiquitylation and proteolysis [8], [9]. Furthermore, FBW7 loss of function has been reported to attenuate p53 activity [10].

The identification of multiple E3 ligases that regulate the function and stability of p53 and c-MYC and the possible involvement of some of these E3 ligases in the regulation of both p53 and c-Myc have raised questions regarding the physiological functions of these E3 ligases. Despite the identification of Pirh2 as an E3 ligase that polyubiquitylates p53 in cell culture assays, its *in vivo* functions remain unknown. Here we report that mice deficient for Pirh2 are viable but display elevated levels of p53 and apoptosis in response to DNA damage. We also demonstrate that c-Myc is a novel Pirh2 interacting protein and that Pirh2 regulates c-Myc expression levels by mediating its polyubiquitylation and proteolysis. Consistent with this novel Pirh2 function, mice mutant for *Pirh2* show elevated levels of c-Myc and increased risk for plasma cell hyperplasia, gammaglobulinemia, and tumorigenesis. In accordance with the role of p53 in suppressing c-Myc oncogenesis, its inactivation considerably increases spontaneous cancer susceptibility of Pirh2 deficient mice. We also report that the expression of PIRH2 is reduced in various human cancers and that lower levels of PIRH2 expression correlate with decreased survival of patients with lung, breast or ovarian cancer.

Collectively, these data support a role for Pirh2 in the *in vivo* regulation of p53 functions, demonstrate its negative regulation of the oncoprotein c-Myc, and support its role as a novel tumor suppressor.

## Results

### Pirh2 Is Dispensable for Development

Although p53 is a substrate for ubiquitylation by several E3-ligases, to date only Mdm2 is known to be required for p53 regulation *in vivo* as its deficiency leads to embryonic lethality in a p53-dependent manner [11]–[13]. To characterize the physiological functions of Pirh2, an E3 ligase previously reported to polyubiquitylate p53 and regulate its stability [14], we generated *Pirh2* mutant mice (Figure S1A and S1B). Western blot analysis indicated loss of Pirh2 protein in *Pirh2^−/−^* cells (Figure S1C). By contrast to Mdm2 deficiency [11]–[13], loss of Pirh2 did not affect embryonic development. *Pirh2^−/−^* mice were born in a Mendelian ratio, were fertile and did not present any apparent developmental defects compared to their *wildtype* (*Wt*) littermates. Analysis of total cell numbers as well as the different cell subpopulations in thymus, spleen, lymph nodes (LN) and bone marrow (BM) of 6 to 8 week-old *Pirh2^−/−^* mice revealed no significant differences compared to *Wt* littermates (Figure S1D and S1E). In addition, [^3^H] thymidine incorporation assay indicated that loss of Pirh2 had no significant effect on the *in vitro* proliferation of T- and B-cells from 6 to 8 week-old *Pirh2^−/−^* mice (Figure S1F). These data indicate that Pirh2 is dispensable for embryonic and postnatal development.

### Pirh2 Regulates p53 Turnover in Response to DNA Damage

**Figure 1:**
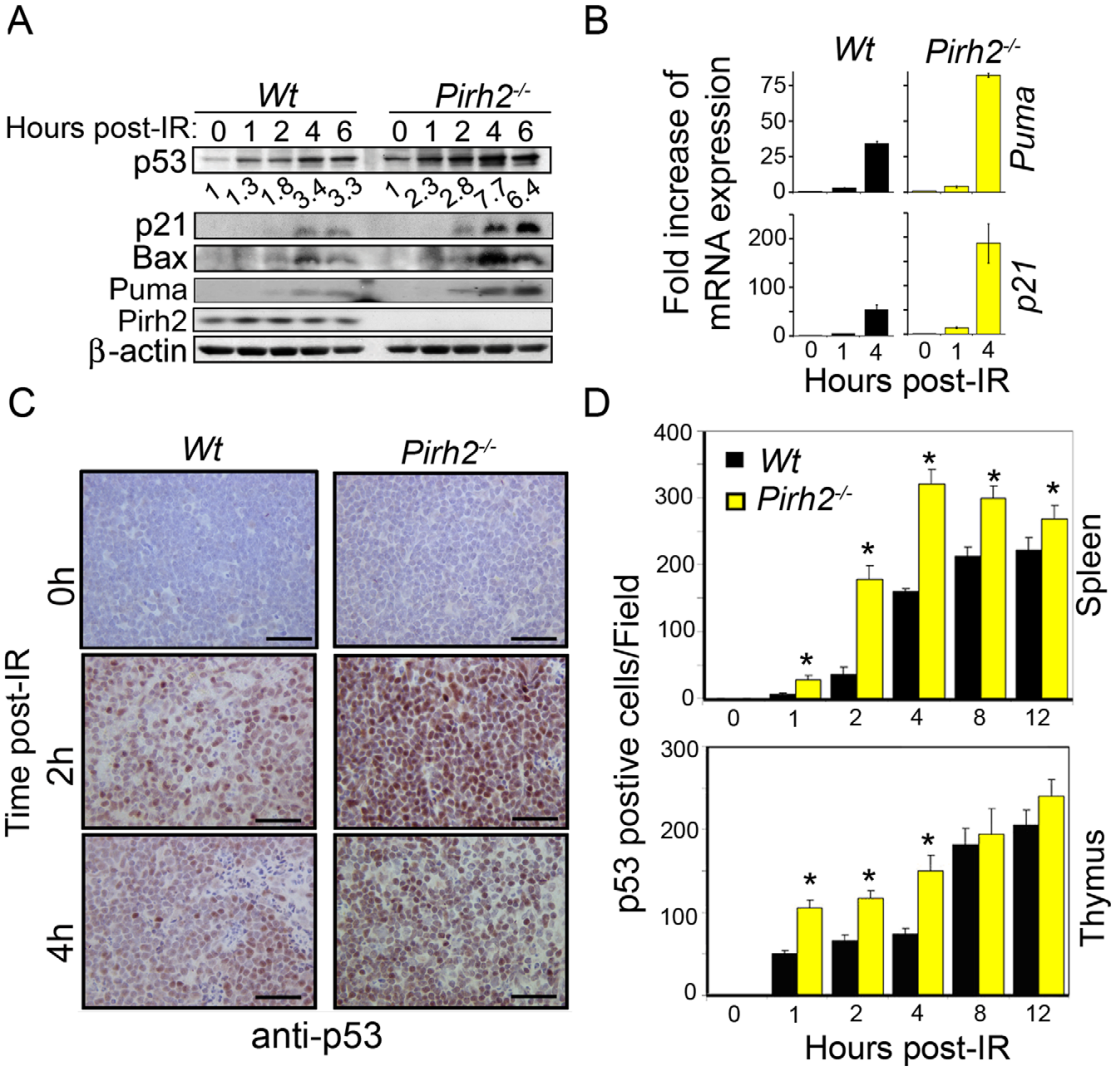
Pirh2 Negatively Regulates p53 Turnover in Response to DNA Damage. (A) Time course immunoblot analysis of the expression of p53, p21, Bax, Puma and Pirh2 in response to irradiation (6 Gγ) of *Wt* and *Pirh2^−/−^* splenocytes. p53 level was quantified based on scanning by densitometry using ImageJ and normalized by β-actin level. p53 fold increase is indicated. Data are representative of four independent experiments. (B) Splenocytes from *Wt* and *Pirh2^−/−^* mice were IR treated (6 Gγ) and their RNA extracted at time 0, 1 and 4 h post-IR. Quantitative RT-PCR analysis was performed to assess gene expression of *Puma* and *p21* and was normalized to *actin* mRNA. Fold changes of mRNA expression in *Wt* and *Pirh2^−/−^* compared to their time 0 h is shown. Student's *t* test was used for statistical analysis. *P*<0.001 for time 0 h *vs.* 1 h or 4 h for *Wt* (n = 4) and *Pirh2^−/−^* (n = 5). Error bars represent SD. (C) *Wt* and *Pirh2^−/−^* 6–8 week-old mice were irradiated (6 Gγ), sacrificed at different times post-IR and the level of p53 expression in spleen was examined by IHC. Data are representative of three independent experiments. (D) p53 positive cells from spleen (upper panel) and thymus (lower panel) of untreated (n = 3) and irradiated (n = 3) mice were counted from 10 different fields for each time point. Student's *t* test was used for statistical analysis. *: *P*<0.05. Error bars represent SD.

p53 is essential for several cellular processes including apoptosis, cell cycle, senescence and metabolism and its stability is regulated by various proteins including the E3 ligases Mdm2, Cop1, and ARF-BP1 [5], [15]. To determine the role Pirh2 plays in the *in vivo* regulation of p53, we examined p53 levels and functions in Pirh2 deficient mice and cells. Splenocytes and thymocytes from *Pirh2^−/−^* and *Wt* mice were irradiated *ex-vivo*, and the levels of p53 expression and activation were determined by Western Blot analysis. Untreated *Pirh2^−/−^* cells displayed mildly increased expression of p53 compared to *Wt* controls (Figure 1A). However, untreated *Pirh2^−/−^* cells, similar to *Wt* controls, displayed no detectable expression of the p53 transcriptional targets *p21*, *bax*, and *Puma* (Figure 1A). We examined the effect of Pirh2 deficiency on irradiation (IR) induced p53 expression and have observed that irradiation of *Pirh2^−/−^* splenocytes resulted in higher expression levels of p53 and its targets *p21*, *bax*, and *Puma* compared to *Wt* controls (Figure 1A and 1B). Consitent with the reported increased transcription of pirh2 in response to p53 activation in mouse embryonic fibroblasts (MEFs) [14], *Pirh2* mRNA was found to increase by 1.5 fold at 1 to 4 h post irradiation of splenocytes (Figure S2A). To examine the *in vivo* effects of Pirh2 loss on the expression of p53, *Pirh2^−/−^* mice and their *Wt* littermates were subjected to whole-body irradiation (6 Gγ) and their tissues were collected at different time points post-irradiation (0–12 h). Immunohistochemistry (IHC) examination of p53 expression indicated that while p53 was undetectable in spleen, thymus, intestinal crypts and liver from untreated *Pirh2^−/−^* and *Wt* mice, the frequency of cells expressing p53 in these organs was significantly higher post-IR of *Pirh2^−/−^* mice compared to *Wt* controls (Figure 1C and 1D, Figure S2B–S2D). We next examined the effect of PIRH2 deficiency on P53 level using human RKO cells in which PIRH2 was knocked down by a tetracycline-inducible system [16]. Similar to murine cells, deficiency of PIRH2 in RKO cells only slightly increased P53 level under untreated conditions, but this increase was more pronounced post-IR (Figure S2E).

Phosphorylation of p53 is critical for its stability and functions [17]. For instance, in response to DNA damage, p53 is phosphorylated on Serine 15 (p53-S15) by a number of kinases including ATM, DNA-PK and ATR and phosphorylation of this p53 site, together with others, is important for its stability and functions [17]. To further examine the effect of Pirh2 deficiency on p53 stability and function, we examined by Western blotting the level of p53-S15 in *Pirh2^−/−^* and *Wt* cells. While, the level of p53-S15 was similarly low in untreated *Pirh2^−/−^* and *Wt* splenocytes, it was significantly higher in irradiated *Pirh2^−/−^* cells compared to *Wt* controls (Figure S3A). Similarly, IHC analysis indicated no differences in the levels of p53-S15 in spleen of untreated *Pirh2^−/−^* mice and *Wt* controls; however a marked increased expression of this phosphorylated form of p53 was observed post whole-body irradiation of *Pirh2^−/−^* mice compared to *Wt* controls (Figure S3B).

We next examined the effect of PIRH2 expression on the level of S15-p53 using H1299 cells transiently transfected with expression vectors for MDM2 or PIRH2 and either wildtype p53, p53 S15A or p53 S15D. The presence of Alanine (A) at residue 15 of p53 prevents p53 phosphorylation at this site while the presence of Asparatic acid (D) at this site mimics its constitutive phosphorylation. Western blot analysis indicated that overexpression of MDM2 or PIRH2 decreased the level of wildtype p53 (Figure S3C). Consistent with previous data, overexpression of MDM2 was able to decrease the stability of p53 S15A and p53 S15D [17]–[19]. Interestingly, while overexpression of PIRH2 resulted in decreased expression of both forms of p53, its effect was more pronounced on p53 S15D. Collectively, these data support a role for Pirh2 in the regulation of p53.

### Pirh2 Deficiency Leads to Elevated Irradiation-Induced Apoptosis

**Figure 2:**
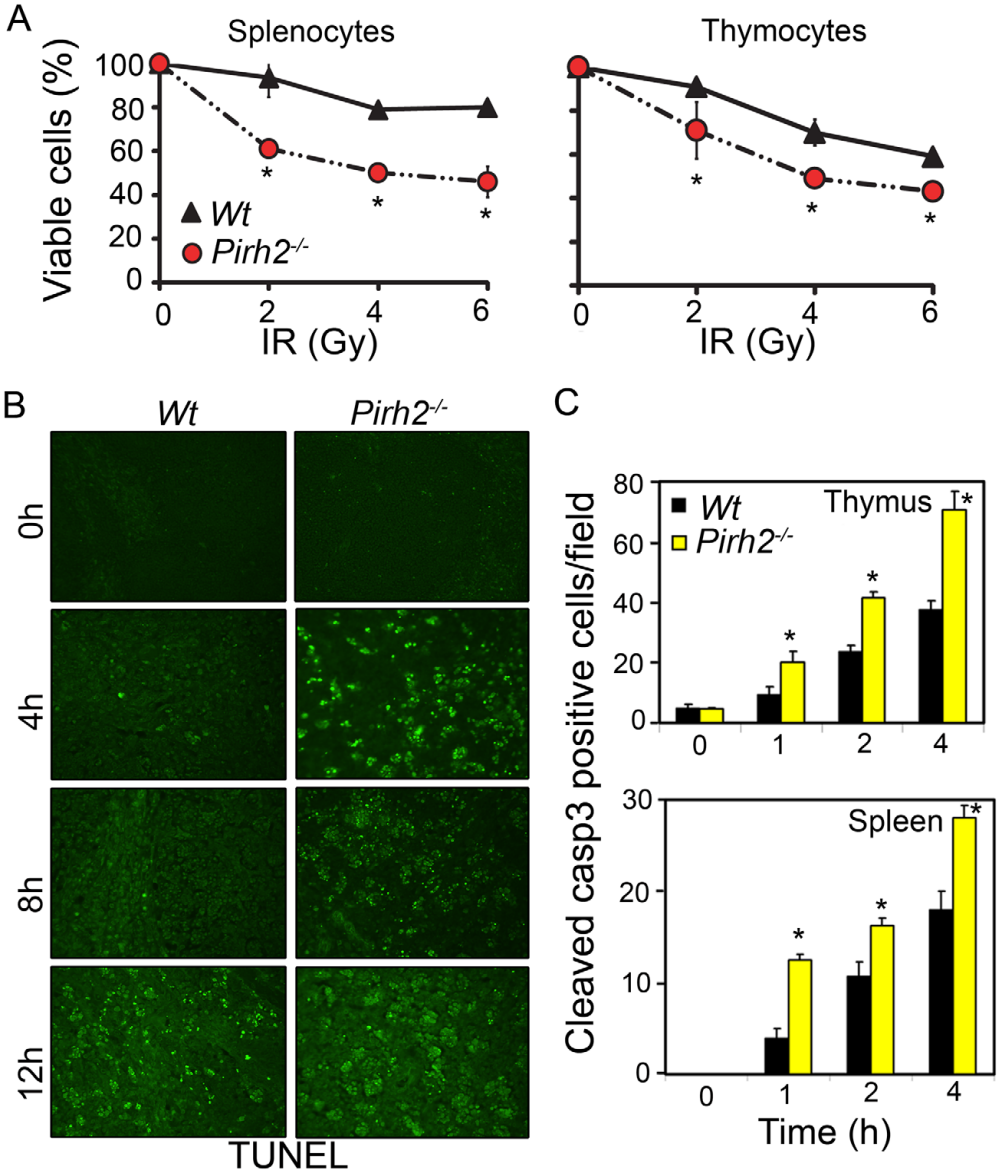
Increased Radiosensitivity in the Absence of Pirh2. (A) Determination of the viability of *Pirh2^−/−^* and *Wt* splenocytes and thymocytes 6 h post *ex vivo* radiation (0–6 Gγ). Viability was determined using Annexin V/PI assay. (B) Time course analysis of apoptosis using TUNEL assay was performed on thymus sections from whole body irradiated (6 Gγ) *Wt* and *Pirh2^−/−^* 6–8 week-old mice. (C) Positive cells for cleaved caspase 3 (casp3) in *Wt* and *Pirh2^−/−^* thymus and spleen sections were counted in 10 different fields for each time point. The data shown in A and C are representative of at least three independent experiments. Student’s *t* test was used for statistical analysis. *: *P*<0.05. Error bars represent SD.

p53 plays central roles in IR-induced apoptosis; therefore we have examined the effect of Pirh2 deficiency on the levels of apoptosis under untreated and DNA damage conditions. Splenocytes and thymocytes from *Pirh2^−/−^* and *Wt* mice were either untreated or irradiated (6 Gγ) and their apoptotic levels examined using the AnnexinV/PI assay (Figure 2A). We also examined the level of apoptosis in thymus, spleen and intestinal crypts from whole-body irradiated (6 Gγ) mice using TUNEL assay and cleaved caspase 3 IHC (Figure 2B and 2C, Figure S3D and S3E). Our data indicated no increased level of spontaneous apoptosis in the absence of Pirh2; however consistent with the elevated IR-induced expression of p53 and its proapototic target genes Bax and Puma in the absence of Pirh2, IR-induced cell death was significantly elevated in *Pirh2^−/−^* cells and mice compared to *Wt* controls. These data demonstrate that Pirh2 deficiency results in elevated IR-induced apoptosis.

### Pirh2 Interacts with the Oncoprotein c-Myc

**Figure 3:**
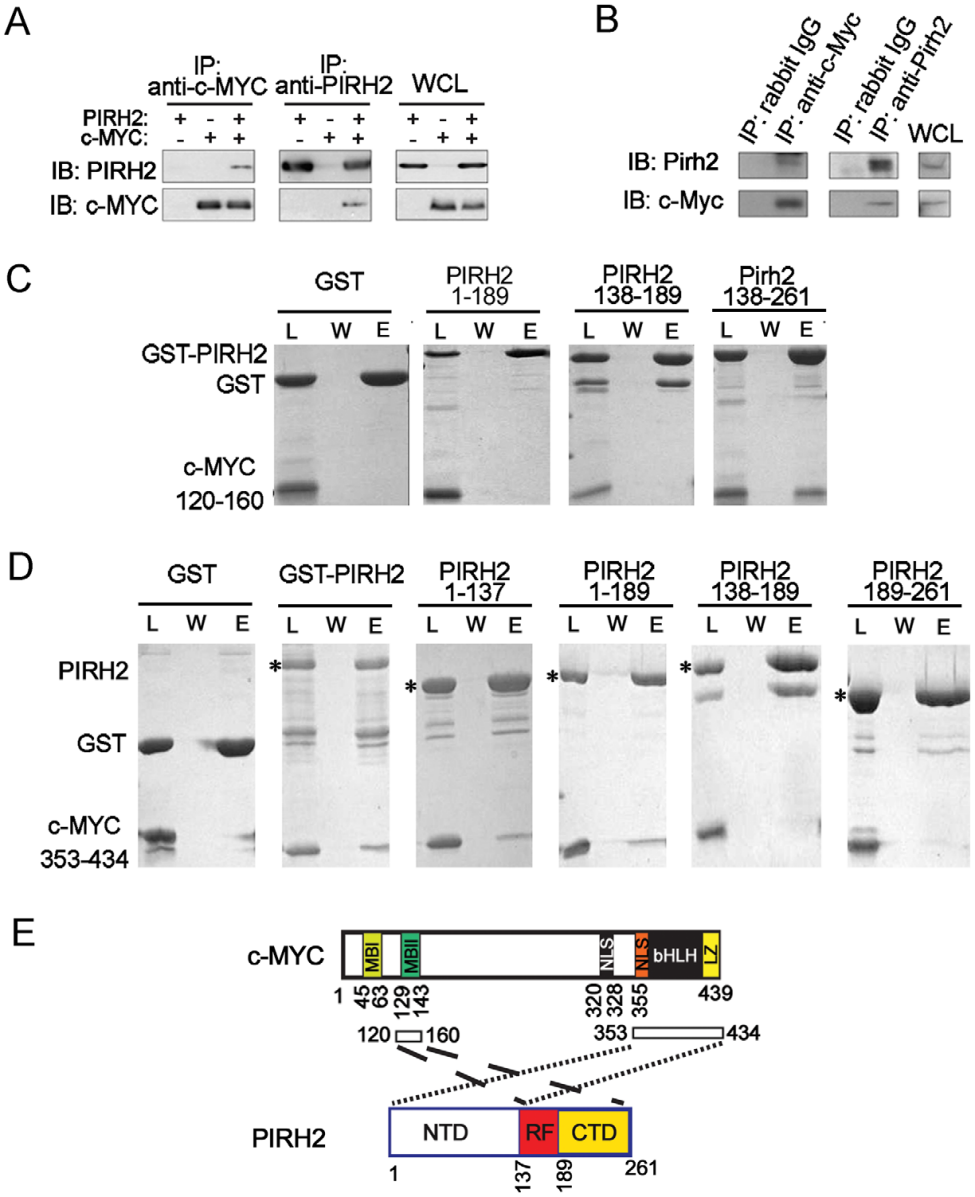
Characterization of the Interaction of PIRH2 and c-MYC. (A) Interaction of human PIRH2 and c-MYC. HEK293T cells were transfected with expression plasmids encoding PIRH2 and c-MYC as indicated. Immunoprecipitation (IP) was performed with anti-PIRH2 or anti-c-MYC antibody and analyzed by immunoblot (IB). (B) NIH3T3 cells were lysed and IP performed with antibodies against murine Pirh2 or murine c-Myc, followed by IB analysis. A portion of the cell lysate corresponding to 3% of the input for IP was subjected to IB analysis. WCL: whole cell lysate. (C) PIRH2 interacts with the N-terminus of c-MYC. GST pull-down assays of GST-PIRH2 fusion proteins with c-MYCboxII (120–160 aa). Labeled lanes reflect loaded material (L), column flow-through after wash (W) and eluate (E). (D) GST pull-down assays of GST-PIRH2 with His-c-MYC (353–434 aa) protein. Star indicates GST-PIRH2 proteins. (E) Schematic representation of the interacting regions of PIRH2 and c-MYC. MBI: c-MYCboxI, MBII: MYCboxII, NLS: nuclear localization signal, bHLH: Basic region and Helix–loop–helix, LZ: leucine zipper.

In our search for novel Pirh2 interacting proteins we identified interaction of Pirh2 and c-Myc. We observed that human PIRH2 and c-MYC reciprocally co-immunoprecipitate upon overexpression in HEK293 cells (Figure 3A). Endogenous interaction of murine Pirh2 and c-Myc was confirmed in NIH3T3 cells where immunoprecipitation of Pirh2 pulled down c-Myc and immunoprecipitation of c-Myc pulled down Pirh2 (Figure 3B).

We next examined whether PIRH2 and c-MYC interaction was direct and characterized the domains involved in this interaction. We performed Glutathione S-transferase (GST) pull-down assays using purified GST or His-fusion proteins of the full length or fragments of PIRH2 and c-MYC. These pull-down assays indicated interaction of PIRH2 amino acids (aa) 138–261 with the N-terminus of c-MYC (aa 120–160) that contains MYCboxII (MBII) but not with N-terminus of c-MYC (aa 1–69) that contains MYCboxI (MBI) (Figure 3C and 3E, Figure S4A). Notably, MBII (aa 128–143) is important for c-MYC proteasomal degradation, transcriptional repression and activation and for controlling c-MYC transformation ability [20]. Pull-down experiments using His-C-terminus of c-MYC (aa 353–434) demonstrated the interaction of this region of c-MYC with the N-terminus of PIRH2 (aa 1–137) (Figure 3D and 3E). These data reveal c-MYC as a novel interacting protein for PIRH2 and indicate the importance of different domains of c-MYC and PIRH2 for this interaction.

### Pirh2 Mediates c-Myc Polyubiquitylation

**Figure 4:**
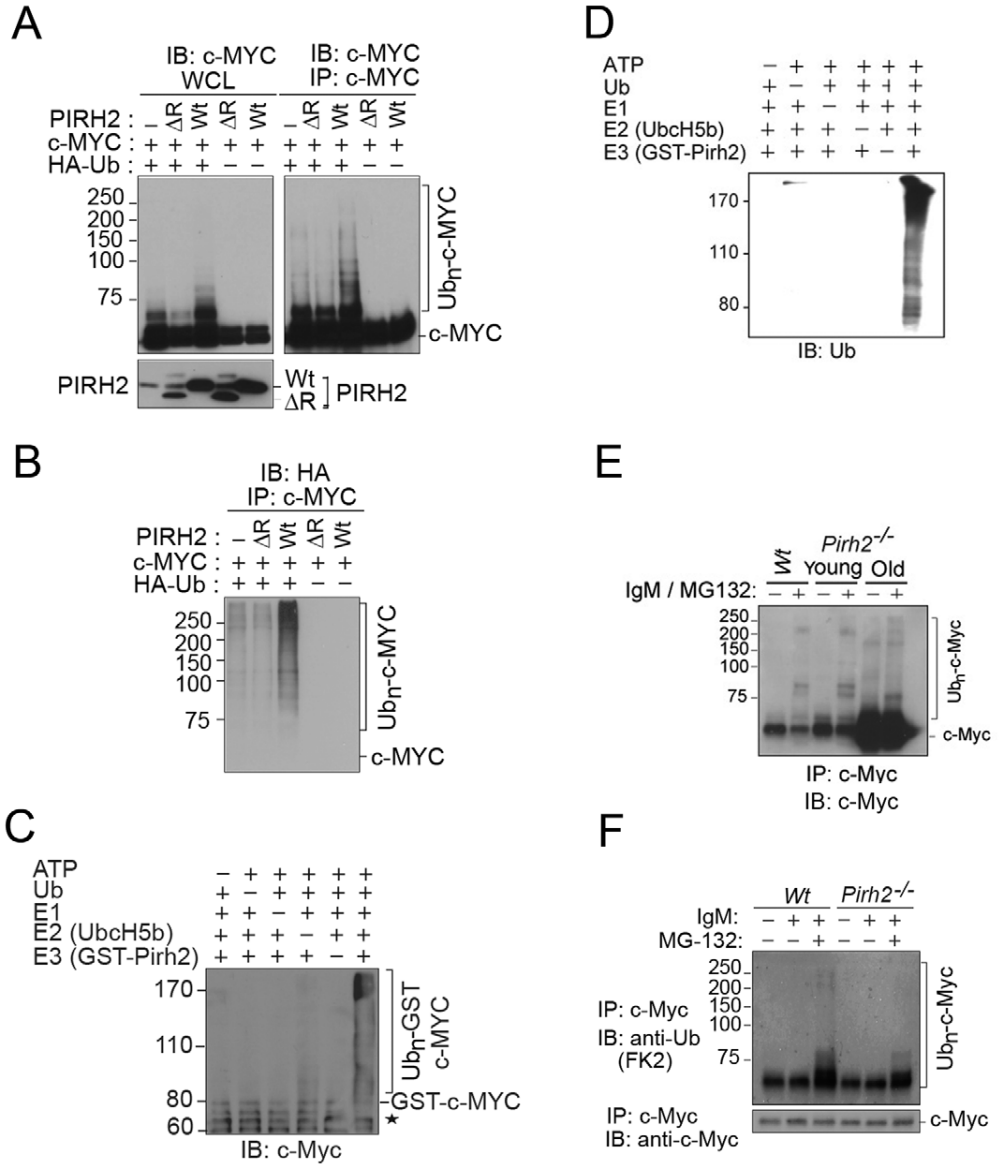
PIRH2 Polyubiquitylates c-MYC and Mediates Its Proteasomal Degradation. (A, B) Intracellular ubiquitylation assay. HEK293T cells were transfected with expression plasmids encoding Wt or deleted RING (ΔR) human PIRH2, c-MYC and HA-tagged ubiquitin (HA-Ub) as indicated. IP using anti-c-MYC antibody were subjected to IB analysis with anti-c-MYC antibody (A) or anti-HA antibody (B). 3% of the input for IP was subjected to IB analysis (A). WCL: whole cell lysate. (C, D) *In vitro* ubiquitylation assay. 6xHis tagged ubiquitin, human ubiquitin activating enzyme E1, UBE2D2/UbcH5b, GST-PIRH2 and GST-c-MYC were used to assess PIRH2 mediated c-MYC ubiquitylation *in vitro*. Ubiquitylated c-MYC proteins were visualized by Western Blot using anti-c-MYC antibody (C) or anti-Ub antibody (U0508) (D). Star indicates GST-c-MYC degradation species. (E) Splenocytes from 8 week-old *Wt* and *Pirh2^−/−^* mice (young) or from 11 month-old *Pirh2^−/−^* mice (old) were cultured in the presence or absence of anti-IgM and/or MG132 for 3 h. Cells were lysed and subjected to IP with anti-c-Myc antibody (N262), and precipitates were subjected to anti-c-Myc (C33) IB analysis. Despite the elevated c-Myc level in samples from *Pirh2^−/−^* mice, the amount of ubiquitylated c-Myc protein remained very low. (F) *In vivo* ubiquitylation assay. Splenocytes were harvested from *Wt* and *Pirh2^−/−^* mice at 8 weeks of age and cultured in the presence or absence of anti-IgM and/or MG132 for 3 h. *Pirh2^−/−^* cell lysates were adjusted to contain an equivalent amount of c-Myc protein compared to *Wt* controls. IP performed with anti-c-Myc antibody was subjected to IB analysis with anti-Ub.

Polyubiquitylation of c-Myc is critical for the regulation of its stability and functions [2], [6]. Our observation that c-MYC interacted with the E3 ligase PIRH2 raised the possibility that it might be a target for PIRH2 mediated ubiquitylation. We examined this hypothesis in HEK293T cells that overexpress HA-Ubiquitin (Ub), human c-MYC, and/or either human full length PIRH2 or a PIRH2 lacking the ring finger domain (PIRH2^ΔR^). 48 h post-transfection, immunoprecipitation followed by Western Blot analysis revealed high-molecular-weight c-MYC species where full length PIRH2, but not PIRH2^ΔR^, were overexpressed in the presence of c-MYC and HA-Ub (Figure 4A). Anti-HA immunoblot of c-MYC immunoprecipitates confirmed that the observed high-molecular-weight c-MYC species in the presence of the full length PIRH2 were indeed due to its polyubiquitylation. Our data also demonstrated the requirement for the RING finger domain of PIRH2 for its polyubiquitylation of c-MYC (Figure 4B). Similarly, we examined the ability of PIRH2 to polyubiquitylate T58A c-MYC as phosphorylation of c-MYC at this site is critical for its polyubiquitylation by FBW7. Consitent with our finding that MBI that contains T58 is dispensible for the interaction c-MYC-PIRH2, PIRH2 was able to ubioquitylate T58A mutant c-MYC (Figure S4B). We also performed *in vitro* ubiquitylation assays and observed that PIRH2 directly polyubiquitylates c-MYC (Figure 4C and 4D).

We next examined the role of Pirh2 in the ubiquitylation of the endogenous c-Myc using *Pirh2^−/−^* and *Wt* B-cells. Anti-IgM activated B-cells were pre-treated for 3 h with the proteasome inhibitor MG132, and c-Myc immunoprecipitates were examined for their levels of ubiquitylation using anti-c-Myc and anti-ubiquitin antibodies. Pirh2 deficiency resulted in reduced level of both high-molecular-weight and polyubiquitylated forms of c-Myc (Figure 4E and 4F). Taken together, these data identified c-Myc as a novel substrate for Pirh2-mediated polyubiquitylation.

### Pirh2 Mediates c-Myc Proteolysis and Its Absence Leads to Accumulation of c-Myc Protein

**Figure 5:**
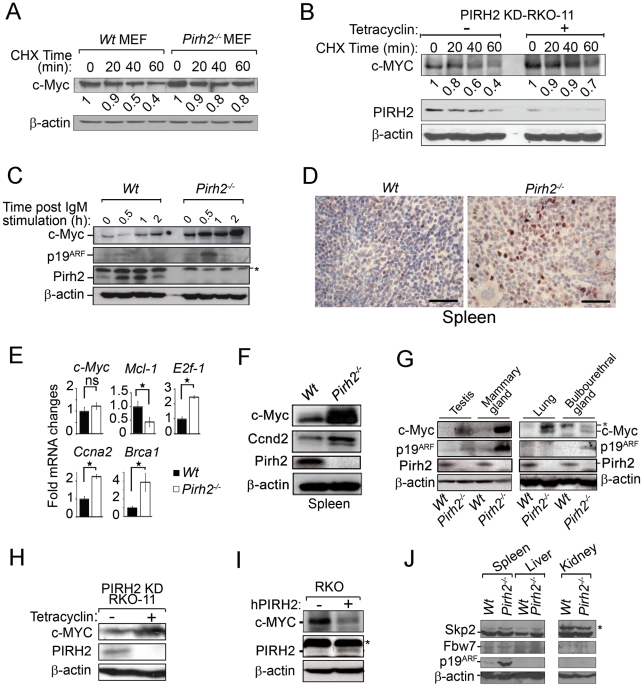
Increased c-Myc Protein Level in *Pirh2* Mutant Cells and Mice. (A) Half-life determination of c-Myc protein in the presence or absence of Pirh2. *Wt* and *Pirh2^−/−^* MEFs were deprived of serum for 48 h, stimulated by re-exposure to serum for 4 h, and then incubated in the presence of CHX for the indicated times. Cell lysates were subjected to immunoblot analysis with antibodies for c-Myc or β-actin. c-Myc level was quantified based on scanning by densitometry using ImageJ and normalized by β-actin level. c-Myc fold increase is indicated. These data are representative of 2 independent experiments. (B) Half-life determination of the level of human c-MYC protein in the presence or absence of PIRH2. Tetracyclin inducible PIRH2 knockdown RKO cells (PIRH2 KD RKO-11) were grown in the absence or presence of tetracyclin for 72 h and CHX was added to these cells for the indicated times. Representative Western blot analysis of the effect of knockdown of PIRH2 on the expression level of c-MYC in RKO cells is shown. c-MYC level was quantified based on scanning by densitometry using ImageJ and normalized by β-actin level. c-Myc fold increase is indicated. (C) Immunoblot analysis of c-Myc, p19^ARF^ and Pirh2 expression in splenocytes from 6 week-old *Pirh2^−/−^* and *Wt* littermates. Cell extracts were prepared at 0, 0.5, 1 and 2 h post anti-IgM stimulation. These data are representative of 4 independent experiments. (D) Sections from *Wt* and *Pirh2^−/−^* spleens were stained with anti-c-Myc antibody (Bar = 50 µm). (E) Quantitative real time PCR was performed to assess the mRNA level of *c-Myc* and its transcriptional targets *E2f1*, *Ccna2*, *Brca1* and *Mcl-1* in spleen from 10 to 13 month-old *Wt* (n = 4) and *Pirh2^−/−^* (n = 12) mice. mRNA expression was normalized to *actin* mRNA. Fold changes of mRNA expression is shown. Student's *t* test was used for statistical analysis. Stars indicate *P*<0.008; ns: not significant. Error bars represent SD. (F) Immunoblot analysis of c-Myc, Ccnd2 and Pirh2 expression in spleen from *Pirh2^−/−^* and *Wt* 10 month-old mice. (G) Immunoblot analysis of c-Myc, p19^ARF^ and Pirh2 expression in testis, mammary glands, lung and bulbourethral glands from 8 week-old *Pirh2^−/−^* and *Wt* mice. *: non specific. (H) Western blot analysis of the expression of c-MYC in tetracyclin inducible PIRH2 knockdown RKO cells (PIRH2 KD RKO-11). Representative Western blots of three independent experiments are shown. (I) Western blot analysis of the effect on c-MYC level of transfected human PIRH2 (hPIRH2) in RKO cells. *: non specific. (J) Representative immunoblot analyses of the level of Skp2, Fbw7 and p19^ARF^ in spleen, liver and kidney from 8 week-old *Wt* and *Pirh2^−/−^* mice. *: non specific.

Since c-Myc polyubiquitylation can control its turnover, we examined the steady state levels of c-Myc in *Wt* and *Pirh2^−/−^* MEFs in the presence of the protein biosynthesis inhibitor cycloheximide (CHX). These studies indicated that while less than 40% of c-Myc remained in *Wt* MEFs 1 h post-CHX treatment, near 80% of c-Myc remained in *Pirh2^−/−^* MEFs (Figure 5A). Similarly, knockdown of human PIRH2 in RKO cells resulted in increased c-MYC stability (Figure 5B).

Because Pirh2 interacted with c-Myc and mediated its polyubiquitylation and proteolysis, we examined the effect of its deficiency on the level of c-Myc protein. We first assessed by Western blotting the expression of c-Myc in B-cells from young (6–8 weeks old) *Pirh2^−/−^* mice and their *Wt* littermates. c-Myc protein level was significantly increased in naïve and in anti-IgM activated *Pirh2^−/−^* B-cells compared to *Wt* controls (Figure 5C). Immunohistochemistry analysis of the expression level of c-Myc protein in spleen from young *Pirh2^−/−^* and *Wt* mice also showed increased c-Myc level in the absence of Pirh2 (Figure 5D). Quantitative real time PCR analysis of cDNA from *Pirh2* ^−/−^ and *Wt* splenocytes indicated that despite the increased protein expression of c-Myc in *Pirh2^−/−^* cells, *c-Myc* mRNA abundance was not significantly affected compared to *Wt* controls (Figure 5E). However, examination of c-Myc transcriptional targets indicated that the RNA expression level of *E2f1*, *Ccna2*, *Ccnd2* and *Brca1* was significantly increased while *Mcl-1* RNA expression was repressed in accordance with the elevated levels of c-Myc in *Pirh2^−/−^* cells (Figure 5E). Increased level of Ccnd2 protein was also observed in splenocytes from young *Pirh2^−/−^* mice compared to *Wt* littermates (Figure 5F). Western blot examination of c-Myc expression in various tissues from young *Pirh2^−/−^* mice and *Wt* littermates indicated increased c-Myc level in the absence of Pirh2, with the highest increase observed in spleen, testis and mammary glands (Figure 5F and 5G).

The tumor suppressor p19^ARF^ has been shown to be upregulated in response to high, but not to low, levels of deregulated c-Myc [21]. Using Western blotting we evaluated the level of p19^ARF^ in cells and tissues from young *Pirh2^−/−^* mice and their *Wt* littermates. The level of p19^ARF^ protein was found elevated in *Pirh2^−/−^* activated B-cells and in tissues that express high levels of c-Myc (e.g. spleen, testis, mammary glands) (Figure 5C, 5G and 5J).

We next examined whether the loss of human PIRH2 would also lead to increased level of c-MYC protein. Western blot analysis indicated that similar to Pirh2 deficiency in mice, knock down of the human PIRH2 in RKO cells significantly increased the protein expression level of c-MYC (Figure 5H). Conversely, overexpression of human PIRH2 in RKO cells downregulated their protein level of c-MYC (Figure 5I).

Since Skp2 and Fbw7 mediate c-Myc polyubiquitylation and proteasomal degradation [2], [6], [22], [23], we examined their expression levels in *Pirh2^−/−^* cells and *Wt* controls. Western blot analyses indicated no difference in the expression levels of Skp2 in spleen, liver and kidney from *Pirh2* ^−/−^ and *Wt* mice, while Fbw7 expression was barely detectable in these organs independently of the genotypes (Figure 5J). We next sought to examine whether PIRH2-c-MYC complex may also contains SKP2 or FBW7. While Western blotting analysis of whole cell lysate of RKO cells showed that these cells expressed PIRH2, SKP2 and FBW7; PIHR2 immunoprecipitation from these cells pulled down c-MYC, but failed to pull down SKP2 or FBW7 (Figure S4C). Therefore SKP2 and FBW7 are not part of the PIRH2-c-MYC complex.

Collectively, these data demonstrate that Pirh2 deficiency leads to increased level of c-Myc protein and that Pirh2 regulates c-Myc stability.

### PIRH2 Expression Is Downregulated in Human Cancer

**Figure 6:**
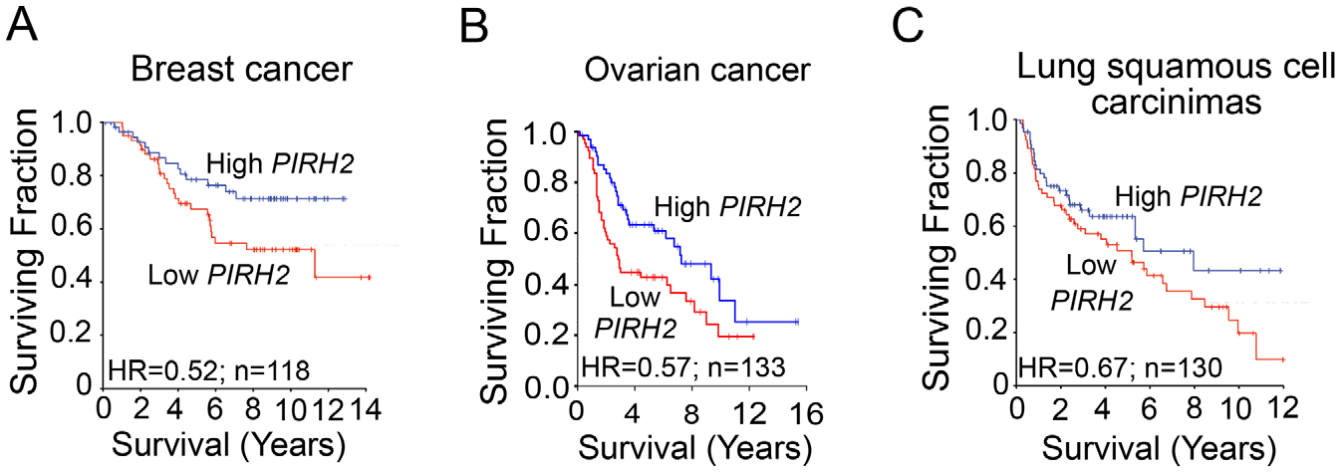
Downregulation of PIRH2 Associates with Short-Term Survival of Patients with Breast Cancer, Ovarian Cancer, or Squamous Cell Carcinomas of the Lung. Datasets of cancer patients at different stages were used to test for any association between *PIRH2* mRNA levels and patient outcome. *PIRH2* mRNA expression is significantly down-regulated in short-term survival breast cancer patients [27] (*P* = 0.049, HR = 0.52, 95% CI 0.27–1.0, n = 118) (**A**), in short-term survival ovarian cancer patients [28] (*P* = 0.02; HR = 0.36–0.92) (B) and short-term survival patients with squamous cell carcinomas of the lung [31] (*P* = 0.11, HR = 0.67, 95% CI 0.41–1.1, n = 130) (C). HR: Hazard ratio.

PIRH2 was reported to be overexpressed in lung and prostate cancer [24], [25]; however, to date no human pathologies have been associated with its decreased expression. To assess whether reduced Pirh2 expression associates with cancer, we examined *PIRH2* expression in primary human cancers using microarray studies that associated mRNA levels to patient outcome. Median dichotomization was used to identify patients with high- and low-*PIRH2* expression levels. We found that lower levels of *PIRH2* mRNA were associated with reduced patient survival in breast cancer [26], [27] (Figure 6A), ovarian cancer [28], [29] (Figure 6B) and squamous cell carcinomas of the lung [30], [31] (Figure 6C).

The expression level of *PIRH2* mRNA was also surveyed in microarray studies of human cancer using the Oncomine database [32]. Expression of *PIRH2* mRNA was significantly reduced in ovarian clear cell adenocarcinoma [33], adult germ cell tumors [34] and invasive bladder cancer [35] compared to controls (Figure S5). These data indicate the high prognostic significance of PIRH2 expression for patients with these tumor types.

### Pirh2 Mutation Leads to Plasma Cell Hyperplasia, Gammaglobulinemia, Kidney Failure, and Premature Death

**Figure 7:**
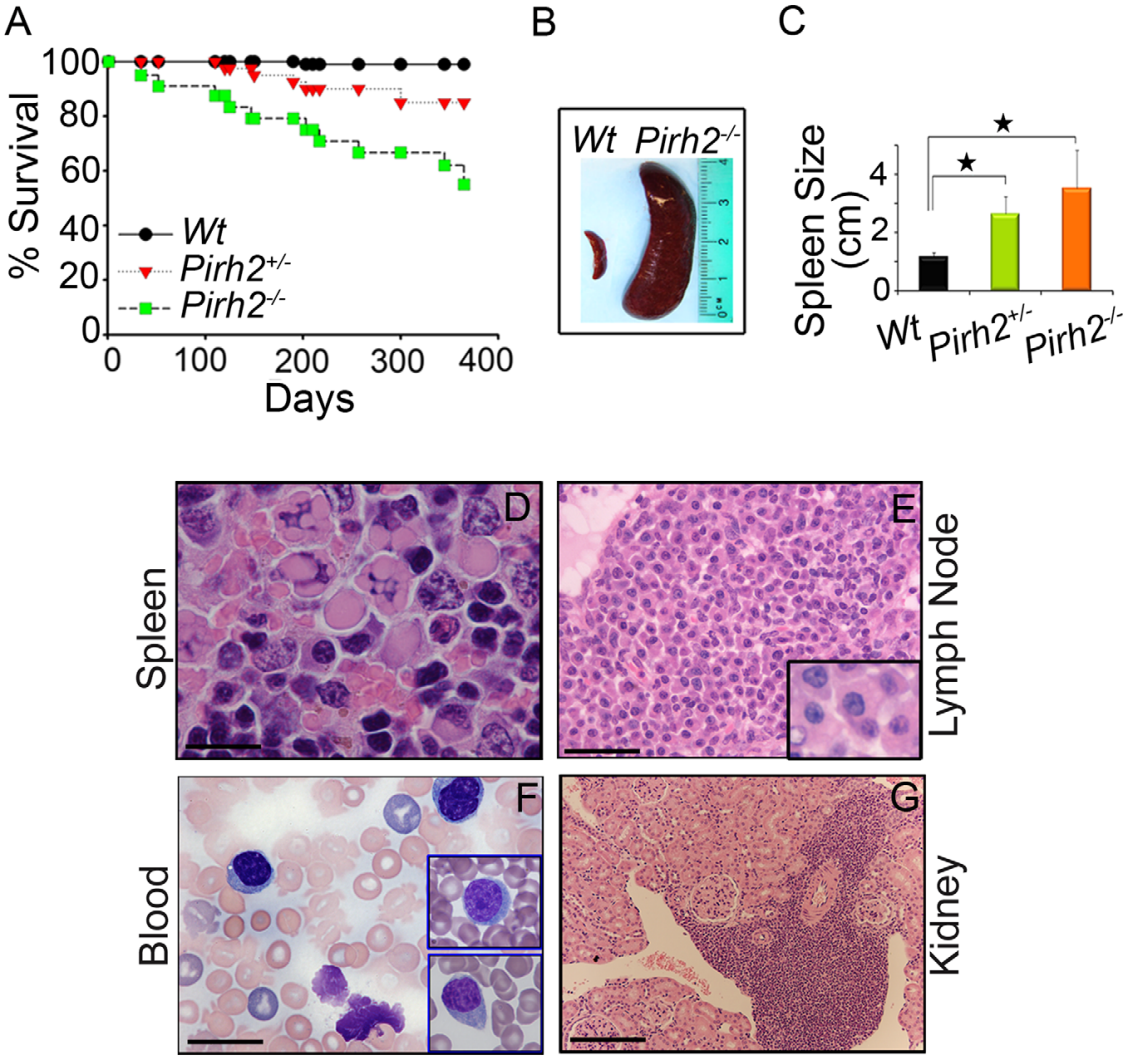
Shorter Life Span and Plasma Cell Hyperplasia of *Pirh2* Mutant Mice. (A) Kaplan-Meier analysis representing the percent survival versus age in days of cohorts of *Wt* (n = 30), *Pirh2^+/−^* (n = 40) and *Pirh2^−/−^* (n = 25) mice. Log-rank test, *P*<0.046 between *Pirh2^+/−^* mice and *Wt* controls; *P*<0.0001 between *Pirh2^−/−^* mice and *Wt* controls. (B) Representative splenomegaly associated with *Pirh2* mutation. (C) Size of spleens from *Wt* (n = 8), *Pirh2^+/−^* (n = 5) and *Pirh2^−/−^* (n = 8) 10 to 12 month-old mice. Student's *t* test was used for statistical analysis. Stars indicate *P*<0.005. Error bars represent SD. (D) H&E staining of *Pirh2^−/−^* spleen sections showing plasma cells with Russell bodies (Bar = 20 µm). (E) H&E staining of sections from grossly enlarged *Pirh2^−/−^* lymph nodes showing focal infiltrates of a large number of plasma cells (Bar = 50 µm; inset: bar = 20 µm). (F) Blood smear performed on *Pirh2^−/−^* mice showing atypical plasma cells (Bar = 20 µm; inset: bar = 20 µm). (G) H&E staining of *Pirh2^−/−^* kidney sections showing plasmacytoid aggregates infiltrating the kidney interstitium (Bar = 100 µm).

Deregulated c-Myc has been associated with a number of diseases including plasma cell hyperplasia, gammaglobulinemia, kidney failure and cancer development [7], [36]–[38]. Monitoring of cohorts of *Pirh2* mutant and *Wt* mice indicated that both *Pirh2^−/−^* and *Pirh2^+/−^* mice had reduced lifespan compared to *Wt* littermates (Figure 7A). Necropsy of *Pirh2* mutant mice indicated that these mutants developed a lymphoproliferative disorder characterized by plasma cell hyperplasia. 100% of sick *Pirh2* mutants presented splenomegaly while lymphadenopathy was observed in 40% and 20% of sick *Pirh2^−/−^* and *Pirh2^+/−^* mice respectively (Figure 7B and 7C). Hematoxylin and Eosin (H&E) staining of spleen and lymph nodes from sick *Pirh2* mutant mice, together with in-touch blood smears, illustrated the presence of plasma cells with large basophilic cytoplasm, eccentric nuclei with clock face appearance and occasional multiple Russell bodies (Figure 7D–7G), all typical features of well differentiated plasma cells. FACS analysis and IHC staining for CD138, a marker for plasma cells, indicated increased proportion of CD138^+^B220^−^ cells in the spleen of sick *Pirh2^−/−^* mice (Figure S6A and S6B). Infiltration of these plasma cells was evident in regional lymph nodes and kidneys (Figure 7E and 7G). *Pirh2^−/−^* plasma cell perivascular infiltrates to non lymphoid organs displayed elevated levels of c-Myc that was accompanied by increased proliferation as indicated by their level of Ki67 (Figure S7A). Consistent with these data and the increased expression of c-Myc in *Pirh2^−/−^* splenocytes, activated T-cells or B-cells from *Pirh2^−/−^* mice displayed higher proliferation rates compared to *Wt* controls (Figure S7B). In addition these *Pirh2^−/−^* activated cells also displayed increased apoptosis compared to *Wt* controls (Figure S7C).

As the cytokine interleukin-6 (IL-6) is important for the differentiation and survival of plasma cells [39], and *Pirh2^−/−^* mice develop a plasma cell disorder, we next examined by ELISA the serum level of IL-6 in *Pirh2^−/−^* mice and their controls. We observed elevated levels of IL-6 in the serum of sick *Pirh2* mutant mice compared to *Wt* littermates (Figure S6D).

Macroscopic analysis of sick *Pirh2^−/−^* and *Pirh2^+/−^* mutant mice indicated the presence of pale kidneys in about 40% of the cases. In order to examine the cause for this kidney defect, H&E staining was performed on kidneys from *Pirh2* mutants and *Wt* littermates. Since infiltration of plasma cells was observed in kidneys from *Pirh2* mutant mice (Figure 7G), we investigated whether the kidney defects of these mice would be associated with immunoglobulin (Ig) deposits. Staining of kidney sections with anti-Ig antibodies indicated the presence of glomerular Ig deposition in *Pirh2^−/−^* and *Pirh2^+/−^* mice but not in age matched *Wt* littermates (Figure S6C).

In accordance with the plasma cell hyperplasia and the elevated level of glomerular Ig deposition observed in *Pirh2* mutant mice, ELISA analysis of serum Ig levels of 10 to 12 month-old *Pirh2^−/−^* mice indicated that these mice suffered gammaglobulinemia. Significantly elevated levels of IgG1, IgG2b and IgA were observed in the serum of *Pirh2^−/−^* mice compared to their *Wt* littermates (Figure S6E).

While plasma cells in sick *Pirh2* mutant mice showed several histological and morphological features of malignancy, their lack of clonality as assessed by rearrangement of Ig loci, and the polyclonal gammaglobulinemia of *Pirh2* mutant mice prevented us from characterizing the disease as a plasma cell neoplasm. These data indicate that Pirh2 deficiency results in plasma cell hyperplasia, gammaglobulinemia, glomerular Ig deposition and kidney failure that likely contribute to the premature death of *Pirh2* mutant mice.

### Pirh2 Is a Tumor Suppressor and It Deficiency Synergizes Tumorigenesis Associated with p53 Loss

**Figure 8:**
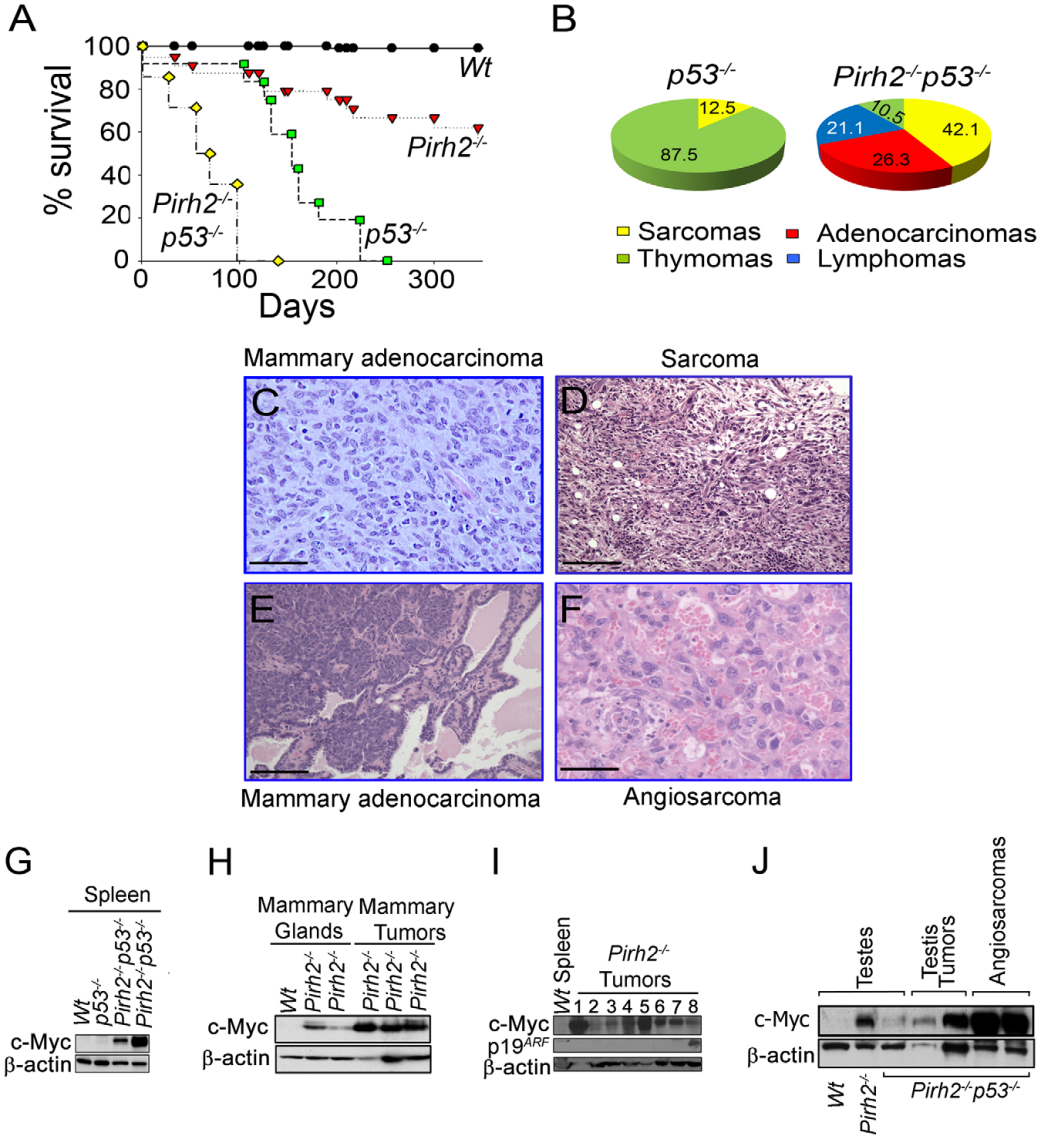
Pirh2 Is a Tumor Suppressor That Collaborates with p53 in Suppressing Cancer. (A) Kaplan-Meier analysis representing the percent survival versus age in days of *Wt* (n = 30), *Pirh2^−/−^* (n = 25), *p53^−/−^* (n = 10) and *Pirh2^−/−^ p53^−/−^* (n = 35) cohort of mice. (B) Tumor spectrum of *p53^−/−^* and *Pirh2^−/−^ p53^−/−^* mice. Log-rank test, *P*<0.0001 between *Pirh2^−/−^ p53^−/−^* mice and *Pirh2^−/−^* or *p53^−/−^* controls. (C) *Pirh2^+/−^*italic> mammary adenocarcinoma (H&E stain; Bar = 100 µm). This tumor is composed of a sheet of pleomorphic epithelial cells with moderate mitotic index, good vascularization and some infiltrating acute inflammatory cells. (D) *Pirh2^−/−^* sarcoma (H&E stain; Bar = 100 µm). This tumor is composed of arrays of fusiform cells, some of them anaplastic; the tumor also shows good vascularity and some lymphocytic infiltration. (E) *Pirh2^−/−^ p53^−/−^* mammary adenocarcinoma (H&E stain; Bar = 500 µm). In contrast with panel C, this tumor is a well-differentiated adenocarcinoma in which acinar structures can be seen interspersed with islets of invasive cells. (F) *Pirh2^−/−^ p53^−/−^* angiosarcomas (H&E stain; Bar = 100 µm). This tumor is mostly constituted by malignant endothelial cells forming irregular spaces filled with red blood cells. (G) Immunoblot analysis showing elevated level of c-Myc in spleen from 6 week-old *Pirh2^−/−^ p53^−/−^* mice compared to *Wt* and *p53^−/−^* controls. (H) Immunoblot analysis showing elevated level of c-Myc in *Pirh2^−/−^* mammary glands from 6 to 8 week-old mice and in *Pirh2^−/−^* mammary tumors. (I) Immunoblot analysis of the level of c-Myc and p19^ARF^ in *Wt* spleen and tumors from *Pirh2^−/−^* mice. 1: sarcoma, 2–6: lung tumors, 7: liver tumor and 8: Breast Tumor tumor. (J) Immunoblot analysis of c-Myc level in testes from 6 week-old *Wt*, *Pirh2^−/−^*, and *Pirh2^−/−^ p53^−/−^* mice and in *Pirh2^−/−^ p53^−/−^* tumors.

Overexpression of ubiquitin ligases including MDM2, COP1 and ARF-BP1 has been observed in human cancer [40]–[42], while downregulation of others such as FBW7 or Rnf8 promotes tumorigenesis [2], [43], [44]. Monitoring of cohorts of *Pirh2* mutant mice indicated that in addition to plasma cell hyperplasia and kidney failure, approximately 25% of *Pirh2^−/−^* and 17% of *Pirh2^+/−^* sick mice developed solid tumors including sarcomas, liver, testes, mammary and lung tumors (Figure 8C and 8D, Figure S8A–S8D and Table S1).

P53 is the most frequently inactivated tumor suppressor in human cancer [45], and its loss facilitates c-Myc induced oncogenesis [46]. To examine the effect of p53 deficiency on development and tumorigenesis of *Pirh2* mutant mice, we crossed *Pirh2^−/−^* and *p53^−/−^* mice and generated *Pirh2^−/−^p53^−/−^* mice. Double mutant mice were born in a Mendelian ratio and showed no developmental defects. Remarkably, *Pirh2^−/−^p53^−/−^* mice had a dramatically reduced lifespan compared to *p53^−/−^* and *Pirh2^−/−^* mice with all double mutants dying before 126 days of age compared to 230 days for *p53^−/−^* littermates (Figure 8A). Necropsy examination of *Pirh2^−/−^p53^−/−^* mice indicated that 60% of them died due to their tumor burden, while the cause of death remained unknown for the remaining mutants. The tumor spectrum of *Pirh2^−/−^p53^−/−^* mice was significantly altered compared to *Pirh2^−/−^* and *p53^−/−^* mutants and consisted of adenocarcinomas, sarcomas, thymomas and T-cell lymphomas (Figure 8B, 8E and 8F; Table S2).

Examination of cells and tissues from healthy *Pirh2^−/−^p53^−/−^* mice indicated that these mice, similar to *Pirh2^−/−^* mice, exhibited elevated levels of c-Myc (Figure 8G and 8J). Western blot analysis also indicated elevated levels of c-Myc in *Pirh2^−/−^* tumors (Figure 8H and 8I); however the majority of these tumors did not show elevated levels of p19^ARF^ (Figure 8I). In addition, similar to *Pirh2^−/−^* tumors, c-Myc levels were also elevated in *Pirh2^−/−^p53^−/−^* tumors (Figure 8J).

These data support Pirh2 function as a tumor suppressor and demonstrate that tumorigenesis of Pirh2 deficient mice is exacerbated by the loss of p53.

## Discussion

Ubiquitylation plays critical roles in the regulation of stability and function of p53 and c-Myc, two of the most important tumor suppressors and oncogenes, respectively. Several E3 ligases have been shown to regulate p53 stability [5]; however, the physiological functions of most of these E3 ligases have not yet been addressed. Mdm2 is well accepted as the master regulator for p53 stability and has been demonstrated for its requirement for embryonic and postnatal development [11]–[13], [47]. Previous studies also indicated that while Mdm2 is required for p53 stability due to its central role in the polyubiquitylation and degradation of p53 [13], [17], Mdm2 independent regulation of p53 degradation also exists. In this study we report that in contrast to Mdm2 deficiency, absence of Pirh2 did not affect embryonic development and only mildly affected p53 steady state levels. However, in response to DNA damage and despite the presence of other E3 ligases for p53 (e.g. Mdm2), Pirh2 deficiency resulted in higher p53 levels in several tissues. Although, we cannot exclude a tissue specificity for the increased IR-induced p53 expression in the absence of Pirh2, collectively our current data indicate a role for Pirh2 in the regulation of p53 stability in response to DNA damage and support a model in which different E3 ligases regulate p53 turnover in a developmental, tissue, stress or time specific manners.

The oncogene c-MYC is frequently deregulated in human cancer [7] and similar to p53, its stability and function are regulated by a number of posttranslational modifications including ubiquitylation. SKP2, FBW7 and ARF-BP1 have been demonstrated to mediate c-MYC ubiquitylation [2], [6]. In this study we identified c-MYC as a novel interacting protein for PIRH2 and demonstrated that this interaction requires both the N- and C-terminal domains of PIRH2 as well as the MBII and the C-terminal domain of c-MYC. Similar to its interaction with PIRH2, c-MYC interaction with SKP2 also involves both its MBII and C-terminal domain [22], [23].

In this study, we also demonstrate that PIRH2 polyubiquitylates c-MYC and that this c-Myc polyubiquitylation controls c-MYC stability and proteolysis. *Pirh2* deficient murine cells and tumors, and human RKO cells knocked down for PIRH2, displayed significantly elevated levels of c-Myc protein, supporting that ubiquitylation control of c-Myc turnover is not only mediated by Skp2 and Fbw7, but also involves Pirh2. Our *in vitro* data showing that PIRH2 interacts with c-MYC and mediates its polyubiquitylation strongly support a direct role for this E3 ligase in c-MYC polyubiquitylation and degradation.

The embryonic lethality of *Fbw7* mutant mice [48] and the postnatal developmental defects of *Skp2* mutants [49], [50] contrast with the normal embryonic and postnatal development observed with *Pirh2* mutants. These developmental differences, together with the differential requirement for these E3 ligases for cancer suppression [2], [43], highlight the complex *in vivo* functions of these E3 ligases.

Although c-Myc protein level was elevated in all examined tissues of *Pirh2^−/−^* mice, spleen, testis and mammary glands displayed the highest levels of c-Myc. In agreement with the upregulation of p19^ARF^ in response to high levels of deregulated c-Myc [21], spleen, testis and mammary glands of *Pirh2^−/−^* mice displayed increased p19^ARF^ expression. However, p19^ARF^ expression was not increased in *Pirh2* ^−/−^ tissues (e.g. lung, liver) that only displayed a moderate elevation of c-Myc expression. As c-Myc induced p19^ARF^ expression results in the sequestration of mdm2 in the nucleolus and the subsequent activation of p53 [51], it is possible that increased levels of c-Myc and p19^ARF^ in *Pirh2^−/−^* mice might contribute to their higher IR-induced p53 responses compared to control littermates. However, our observation that liver of *Pirh2^−/−^* mice display elevated IR-induced p53 expression compared to Wt controls whithout any detectable level of p19^ARF^, supports that increased IR-induced p53 expression in *Pirh2* mutants can take place independently of p19^ARF^. Further biochemical and genetic studies are required to determine whether the increased expression of c-Myc and p19^ARF^ in Pirh2 deficient cells affects their p53 responses.

Our finding that Pirh2 polyubiquitylates c-Myc and mediates its proteolysis, and the elevated c-Myc level associated with Pirh2 deficiency support the possibility that deregulated c-Myc in *Pirh2^−/−^* mice may increase their cancer susceptibility. Indeed, *Pirh2* mutant mice displayed increased predisposition to develop tumors including sarcomas, testis, mammary and lung tumors. In accordance with the role p53 plays in suppressing c-Myc induced oncogenesis [46], *Pirh2^−/−^p53^−/−^* mice showed a markedly increased cancer predisposition compared to single mutants. The accumulation of c-Myc in *Pirh2* mutant mice is also consistent with their elevated risk for developping plasma cell hyperplasia, gammaglobulinemia and kidney failure [36]–[38].

Human PIRH2 has been reported to be overexpressed in lung and prostate cancer [24], [25]. However, the human *PIRH2* locus at 4q21 is lost in a diverse subset of solid tumors including epithelial tumors of the breast and lung [52], [53]. We report in this study that *PIRH2* expression is downregulated in various human cancers and that lower PIRH2 expression correlates with decreased survival of patients with lung squamous cell carcinomas, breast or ovarian cancer. Interestingly, Pirh2 mutant mice also displayed increased risk for lung and breast cancer.

The ability of the E3 ligase Pirh2 to negatively regulate IR-induced p53 and c-Myc steady state level, together with the increased risk for *Pirh2* mutant mice to develop various pathologies, including cancer, all highlight the importance of this novel tumor suppressor and demonstrate the requirement for its tight regulation.

## Materials and Methods

### Generation Pirh2 Mutant Cells and Mice

E14K embryonic stem cells were electroporated with the linearized targeting construct for Pirh2 (Figure S1) and Pirh2^fl2-3-neo^ ES clones identified by PCR and Southern blotting. Pirh2^Δ2-3^ ES clones lacking exon 2 and 3 and the Neomycin resistance cassette were obtained following transient transfection of two targeted Pirh2^fl2-3-neo^ ES clones with CMV-Cre recombinase. Pirh2^Δ2-3^ ES clones were used to derive two independent lines of mutant mice (referred here as Pirh2 mutants). All mice were in 129/C57BL/6 genetic background and were maintained in the animal facility of the Ontario Cancer Institute in accordance with the established ethical care regulations of the Canadian Council on Animal Care. P53 mutant mice [54] were obtained from Taconic.

MEFs were derived according to standard procedures. HEK293T cells, RKO cells and PIRH2-KD RKO clone #11 in which PIRH2 can be knocked down by a tetracycline-inducible system [16], have been also used.

### Flow Cytometry

Single cell suspensions from thymi, spleens, lymph nodes and bone marrow of Wt and Pirh2 mutant mice were stained with the following antibodies against the cell surface markers: CD4, CD8, CD3, CD43, CD95, CD138, IgM, IgD, B220, and Thy1.2 (ebioscience and Pharmingen). Stained cells were analyzed by flow cytometry using the FACScan and Cellquest software.

### Cell Activation Assay

Activation of T and B-cells was performed as previously described [55].

### Quantitative Real Time PCR and Genomic PCR

Cells were harvested and total RNA isolated with Trizol (Gibco). cDNA was generated using Invitrogen kit. SYBR green kit from Applied Biosystems was used for Real-time PCR. The following oligonucleotides were used: *Actin* (5′AACAGGAAGCCCATCACCATCTT3′ and 5′GCCCTTCCACAATGCCAAAGTT3′), *IL-6* (5′TAGTCCTTCCTACCCCAATTTCC3′ and 5′TTGGTCCTTAGCCACTCCTTC3′), *c-Myc* (5′ATGCCCCTCAACGTGAACTTC3′ and 5′CGCAACATAGGATGGAGAGCA3′), *Ccna2* (5′GCCTTCACCATTCATGTGGAT3′ and 5′TTGCTGCGGGTAAAGAGACAG3′), *Ccnd2* (5′GAGTGGGAACTGGTAGTGTTG3′ and 5′CGCACAGAGCGATGAAGGT3′), *E2f1* (5′CAGAACCTATGGCTAGGGAGT3′ and 5′GATCCAGCCTCCGTTTCACC3′), *Mcl-1* (5′AAAGGCGGCTGCATAAGTC3′ and 5′TGGCGGTATAGGTCGTCCTC3′), p21 (5′CCTGGTGATGTCCGACCTG5′ and 5′CCATGAGCGCATCGCAATC3′), *Puma* (5′CCTGGGTAAGGGGAGGAGT3′ and 5′AGCAGCACTTAGAGTCGCC3′), *Brca1* (5′AAGGAGCCCGTGTGCTTAG3′ and 5′TTGCCCTAGATGTGTTGTCTTTT3′), and *Pirh2* (5′CAGACTTGTGAAGACTGTAGCAC3′ and 5′ CGAAGATTCGTGGTTAGGCAT3′). PCR were performed on the Applied Biosystem 7900HT Fast Real-Time PCR system.

### GST Pull-Down Assays

Recombinant purified full length PIRH2 and its fragments were incubated with His-c-MYC fusion proteins in an assay buffer containing PBS (pH 7.4), 100 mM NaCl, 1 mM Benzamidine, 0.5 mM PMSF and 5 mM βME in a 1∶2 molar ratio at 4°C overnight. Then, proteins were incubated with the 100 µl of glutathione-Sepharose beads (GE Healthcare) for an additional 2 h. The mixture was transferred to a microcolumn and was extensively washed with the assay buffer. Bound proteins were eluted with 30 mM reduced glutathione and detected by SDS-PAGE and Coomassie staining

### Western Blot and Immunoprecipitation Analysis

Western blot analysis was performed as previously described [55]. The following antibodies were used: c-Myc (C33 and N262; Santa Cruz), Ccnd2 (Santa Cruz), PIRH2 (Santa Cruz and BETHYL laboratories), SKP2 (Santa Cruz), FBW7 (Sigma), p19^ARF^ (Novus and Calbiochem), and anti-β-actin (Sigma).

HEK293T cells were transfected with expression plasmids encoding human PIRH2 (pcDNA3-hPIRH2) and c-MYC (pcDNA3-c-MYC) using the calcium phosphate method. After 48 h, the cells were lysed in a solution containing 50 mM Tris-HCl (pH 7.4), 150 mM NaCl, 1% Triton-X, 1 mM phenylmethylsulfonyl fluoride, 400 µM Na_3_VO_4_ and protease inhibitor cocktail tablet (Roche). The cell lysates from transfected HEK293T and non transfected NIH3T3 were centrifuged at 14,000×g for 10 min at 4°C, and the resulting supernatant was incubated with anti-c-MYC antibody (N-262) for 2 h at 4°C. Protein G-Sepharose (Amersham) was added to the mixture and the sample rotated overnight at 4°C. The resin was separated by centrifugation, washed four times with ice-cold lysis buffer, and then boiled in SDS sample buffer. Immunoblot analysis was performed with the anti-hPIRH2 (BETHYL laboratories) or anti-c-MYC antibody (mouse monoclonal antibody; 9E10), horseradish peroxidase-conjugated antibodies to mouse or rabbit immunoglobulin G (1∶10,000 dilutions, Cell Signaling) and an enhanced chemiluminescence system (ECL, Amersham). Immunoprecipitation of endogenous PIRH2 from RKO cells was performed using anti-PIRH2 antibody from Santa Cruz (FL261). pcDNA3-hPIRH2 was transfected in RKO cells using the calcium phosphate method.

### Intracellular Ubiquitylation Assay

HEK293T cells were transfected with expression plasmids encoding the full length or a mutant lacking the ring finger domain (ΔR) of human PRIH2 together with Wt or T58A c-MYC and HA-tagged ubiquitin by the calcium phosphate method. After 48 h, the cells were lysed in a modified RIPA buffer as described earlier. The cell lysates were centrifuged at 14,000×g for 10 min at 4°C, and the resulting supernatant was incubated with anti-c-MYC antibody (N262) for 2 h at 4°C. Protein G-Sepharose was added to the mixture, which was then rotated overnight at 4°C. The resin was separated by centrifugation, washed four times with ice-cold lysis buffer, and then boiled in SDS sample buffer. Immunoblot analysis was performed with the anti-ubiquitin (FK2; BIOMOL international) or anti-c-MYC antibody (9E10), horseradish peroxidase-conjugated antibodies to mouse or rabbit immunoglobulin G and ECL.

### *In Vivo* Ubiquitylation Assay

Splenocytes from *Wt* and *Pirh2^−/−^* 8 week-old mice were cultured in the presence or absence of anti-IgM (20 µg/ml) and/or MG132 (20 µM; calbiochem) for 3 h. Cells were then lysed in a modified RIPA buffer as described earlier and cell lysates immunoprecipitated with anti-c-Myc antibody (N262). The immunocomplexes were denatured in Laemmli's sample buffer (25 mM Tris pH 8.3, 192 mM glycine, 0.1% SDS) to dissociate contaminant proteins associated with c-MYC. c-MYC proteins were reimmunoprecipitated (2^nd^ IP) with anti-c-Myc antibody. The resin was separated by centrifugation, washed four times with ice-cold lysis buffer, and then boiled in SDS sample buffer. Immunoblot analyses were performed with the anti-ubiquitin (FK2) or anti-c-Myc antibody (9E10), horseradish peroxidase-conjugated antibodies to mouse immunoglobulin G and ECL.

### *In Vitro* Ubiquitylation Assay

Human ubiquitin activating enzyme E1 and 6xHis tagged ubiquitin were purchased from Boston Biochem. The ubiquitylation reaction was performed in a volume of 20 µL in a buffer of 50 mM Tris pH 7.6, 5 mM MgCl_2_, 2 mM ATP, 2 mM DTT. The reaction mixture typically contained E1 (50 ng, Calbiochem), UBE2D2/UbcH5b (100 ng), Ubiquitin (5 µg, Sigma), Pirh2 (1 µg) and full-length c-Myc (0.5 to 1 µg). After incubation at 30°C for 1.5 h, the reactions were stopped by addition of SDS-PAGE sample buffer and resolved on 7.5–10% SDS-PAGE gels. Ubiquitylated proteins were visualized and evaluated by Western Blot using anti-c-MYC (N262) and anti-Ub antibody (U0508, Sigma).

### Immunohistochemistry

Tissues and tumors fixed in buffered formalin were processed for paraffin-embedded sectioning at 5 µm, and stained with H&E (Fisher). IHC was performed using anti-c-Myc (C19, Santa Cruz), anti-cleaved caspase 3 (Cell Signaling) or Ki67 (Dako). TUNEL staining was performed using an *in situ* cell death detection kit (Boehringer Mannheim).

### Microarray Data Analysis

Microarray data from public studies were downloaded from GEO [26]–[30]. All data was log_2_ transformed and normalized according to the original authors, with the exception of the lung adenocarcinoma dataset, whose pre-processing has been described elsewhere [56]. Unadjusted Cox proportional hazards models were fit in the R statistical environment (v2.6.2) using the survival package (v2.34). P-values were calculated using the Wald test. Correlation analyses used Pearson's correlation as implemented in R (v2.6.2). Scatter- and box-plots were generated using the lattice package (v0.17–4). Long-term (>7 years) vs. short-term survival groups in ovarian cancer dataset [29] were analyzed using a t-test with Welch's adjustment for heteroscedascity. We have used all 25 long-term and all 30 short-term survival samples available from the raw data. Analyzing only strictly long- and strictly short-term survival samples (n = 23 and n = 27 respectively) does not change the average expression and standard error; it only reduces the significance to p = 0.002162. Additional data were considered from Oncomine Research Edition v.3.6, with statistical analysis as described previously [32].

## Supporting Information

Figure S1 *Pirh2^−/−^* mice display normal immune cell development and proliferation of T and B-cells. (A) A schematic representation of the *Pirh2* locus, the targeting construct, and the mutant *Pirh2* alleles (*Pirh2^fl2-3-neo^*, *Pirh2^fl2-3^* and *Pirh2^Δ2-3^*). Exons are indicated as boxes, *loxP* sites as triangles and positions of 5′ and 3′ probes used for Southern blot are indicated. S, *Sst1* site. Expected sizes of *Sst1* DNA fragments are indicated. (B) Southern blot analysis of *Sst1* digested tail genomic DNA of a litter from *Pirh2^+/−^* intercrosses. 5′ probe shown in (A) was used. (C) Western blot analysis of cell extracts from *Wt* and *Pirh2^−/−^* splenocytes showing loss of Pirh2 expression in mutant cells. (D) A representative FACS analysis of thymus, spleen, LN and BM cells from 8 week-old *Wt* and *Pirh2^−/−^* mice. Percentages of populations are indicated. (E) Thymocyte and splenocyte numbers from 6 to 10 week-old *Wt* (n = 6), *Pirh2* ^+/−^ (n = 5) and *Pirh2^−/−^* (n = 9) mice. No significant differences were observed between *Wt* and mutant mice. (F, left panel) T-cells from *Wt*, *Pirh2* ^+/−^ and *Pirh2^−/−^* 6 to 10 week-old mice were activated using anti-CD3 and IL2 and their level of proliferation determined using [^3^H] Thymidine incorporation assay. Data for 48 h and 72 h time points are shown and are representative of 5 independent experiments. (F, right panel) B-cells from *Wt*, *Pirh2* ^+/−^ and *Pirh2^−/−^* mice were activated with anti-IgM+IL4, anti-IgM+CD40, or with LPS and their proliferation determined using [^3^H] Thymidine incorporation assay. Data for 48 h time point are shown. This result is representative of 5 independent experiments. UT = untreated. BM: bone marrow.
(TIF)

Figure S2 Pirh2 deficiency leads to higher accumulation of p53 in response to irradiation. (A) Splenocytes from *Wt* and *Pirh2^−/−^* mice were IR treated (6 Gγ) and their RNA extracted at time 0, 1 and 4 h post-IR. Quantitative RT-PCR analysis was performed to assess *Pirh2* expression and was normalized to *actin* mRNA. Fold changes of *Pirh2* mRNA expression in irradiated *Wt* splenocytes compared to their untreated controls (time 0 h) is shown. Student's *t* test was used for statistical analysis. *P<0.05 compared to time 0 h. *Wt* (n = 4) and *Pirh2^−/−^* (n = 5). Error bars represent SD. (B, C) 6 to 8 week-old *Wt* and *Pirh2^−/−^* mice either untreated or 2 h post whole-body irradiation (6 Gγ) were sacrificed and IHC was performed to assess the level of p53 in thymus (B) and intestinal crypts (C). Bar = 50 µm. (D) p53 positive cells in intestinal crypts (left) and liver (right) of untreated (n = 3) and irradiated (n = 3) *Wt* and *Pirh2^−/−^* mice were counted from 10 different fields for each time point. Student's *t* test was used for statistical analysis. * *P*<0.0005. Error bars represent SD. (E) A representative Western blot of three independent experiments showing the expression of p53 following tetracyclin induced PIRH2 knockdown in the human RKO cells.
(TIF)

Figure S3 Absence of Pirh2 leads to higher levels of Serine 15 phosphorylated p53 and increased apoptosis in response to irradiation. (A) Time course immunoblot analysis of the expression level of Serine 15 phosphorylated p53 (S15-p53), total p53, Bax and Pirh2 in response to irradiation (6 Gγ) of *Wt* and *Pirh2^−/−^* splenocytes. *: non specific. (B) 6 to 8 week-old *Wt* and *Pirh2^−/−^* mice either untreated or 1 h post whole-body irradiation (6 Gγ) were sacrificed and IHC was performed to assess the level of S15-p53 in their spleen. Bar = 50 µm. (C) H1299 cells were cotransfected with either pcDNA3.1, pcDNA3.1-Mdm2 or pcDNA3.1-PIRH2 and a p53 expression vector (Wt, S15A, S15D). Lysates prepared 40 h post-transfection were examined by Western blotting using the indicated antibodies. (D) 6 to 8 week-old *Wt* (n = 3) and *Pirh2^−/−^* mice (n = 3) were subjected to whole-body irradiation (6 Gγ) and the levels of apoptosis in thymus at different time points post-IR were examined using active caspase 3 (casp3) and IHC. Bar = 100 µm. (E) Active casp3 positive cells in intestinal crypts of untreated (0 h) and irradiated *Wt* (n = 3) and *Pirh2^−/−^* (n = 3) mice were counted from 10 different fields for each time point. Student's *t* test was used for statistical analysis. * *P*<0.005. Error bars represent SD.
(TIF)

Figure S4 PIRH2 does not interact with SKP2 or FBW7 and ubiquitylates c-MYC independently of its phosphorylation at T58. (A) PIRH2 does not interact with c-MYCbox I (MBI). GST pull-down assays of GST-PIRH2 fusion protein with His-c-MYC boxI (1–69 aa) protein. Labeled lanes reflect loaded material (L), column flow-through after wash (W) and eluate (E). (B) Intracellular ubiquitylation assay. HEK293T cells were transfected with expression plasmids encoding Wt human PIRH2, HA-tagged ubiquitin (HA-Ub), c-MYC Wt or c-MYC T58A as indicated. IP using anti-c-MYC antibody were subjected to IB analysis with anti-c-MYC antibody (left panel). 3% of the input for IP was subjected to IB analysis with anti-cMYC and anti-PIRH2 (right panel). WCL: whole cell lysate. (C) Representative IP/Western blot data for three independent experiments showing that IP of PIRH2 from human RKO cells pulls down c-MYC but not SKP2 or FBW7. IP: immunoprecipitation. WCL: whole cell lysate.
(TIF)

Figure S5 Reduced levels of *PIRH2* mRNA in human cancers. (Left panel) Data [33] provided by Oncomine Research Edition v.3.6 [32] show significant downregulation of *PIRH2* mRNA in ovarian clear cell adenocarcinoma compared to normal ovary (^*^ *P* = 5.1×10^−6^; t-test). (E, Middle panel) Data [34] provided by Oncomine Research Edition v.3.6 show significant downregulation of *PIRH2* mRNA in adult germ cell tumors compared to normal testis (^*^ *P* = 1.7 10^−18^; t-test). (E, Right panel) Data [35] provided by Oncomine Research Edition v.3.6 show significantly downregulated *PIRH2* mRNA level in invasive compared to superficial bladder cancer (^*^ *P* = 4.5 10^−5^; t-test).
(TIF)

Figure S6 Accumulation of CD138^+^B220^−^ cells, glomerular immunoglobulin deposition and elevated serum immunoglobulins in *Pirh2* mutant mice. (A) Cells from spleen of 11 month-old *Wt and Pirh2^−/−^* littermates were stained with anti-CD138 (a marker for normal and malignant plasma cells), anti-B220 (a marker for B-cells) and FACS analysis was performed. The percentage of CD138^+^B220^−^ cells is indicated. (B) Liver sections from a 10 month-old *Pirh2^−/−^* mouse were stained with anti-CD138. A sheet of CD138^+^ cells infiltrating the liver is shown. Bar = 35 µm. (C) Glomerular Immunoglobulin deposition in 10 to 12 month-old *Pirh2^−/−^* and *Pirh2^+/−^* mice. Immunoglobulin deposits in kidneys from the autoimmune *Lpr* mice are show as positive controls. (D) Elevated level of IL-6 in the serum of 10 month-old *Pirh2^−/−^* mice compared to *Wt* littermates. Student's *t* test was used for statistical analysis. *P<0.005. Error bars represent SD. (E) ELISA analysis of the level of IgG1, IgG2b and IgA serum Ig in 10 to 12 month-old *Pirh2^−/−^* and *WT* mice. * *P*<0.05. Bar = 50 µm.
(TIF)

Figure S7 Plasma cell infiltration to non lymphoid organs of *Pirh2^−/−^* mice. IHC of the lung of a *Pirh2^−/−^* mouse showing structurally normal lung with perivascular plasma cell infiltrates. The infiltrates stain positive for c-Myc and Ki67. Lung section of *Wt* littermates are shown as controls. Left panels: bars = 500 µm. Right panels: bars = 35 µm. (B) FACS analysis of CFSE dilution profiles. LPS induced proliferation of CFSE-labeled B-cells (top panel) and Anti-CD3 induced proliferation of CFSE-labeled T-cells (lower panel) were examined 72 h post activation. Data are representative of four independent experiments. (C) Cell death of cells described in panel B was determined using AnnexinV/PI staining 72 h post-activation. Student's *t* test was used for statistical analysis. *: *P*<0.005. Error bars represent SD.
(TIF)

Figure S8 Tumorigenesis of *Pirh2* mutant mice. (A, B) H&E staining of adenosquamous cell mammary carcinoma from a *Pirh2^−/−^* mouse. This tumor is composed of islets of malignant epithelial cells, with invasion to surrounding connective tissue; keratin pearls are evident within the tumor (panel A: upper and lower left corners). Higher magnification shows the glandular (panel B: lower right) and squamous cellular phenotypes (panel B: upper left and right). (A, Bar = 500 µm; B, Bar = 100 µm). (C, D) H&E staining of a *Pirh2^−/−^* lung neoplasm showing a solitary nodular mass with a well-differentiated adenomatous pattern. This lesion has well-defined borders, is highly vascularized and is surrounded by normal lung with occasional mononuclear inflammation foci. (C, Bar = 500 µm; D, Bar = 100 µm).
(TIF)

Table S1 Plasma Cell Hyperplasia and Tumor Development in *Pirh2* Mutant Mice.
(PDF)

Table S2 Tumor types in *Pirh2^−/−^p53^−/−^* Mutant Mice.
(PDF)
