# Supplementary figures and images for: Role of Pirh2 in Mediating the Regulation of p53 and c-Myc
Source: PLoS Genet. 2011 Nov 17;7(11):e1002360. doi: 10.1371/journal.pgen.1002360 (PMC3219591; doi:10.1371/journal.pgen.1002360)

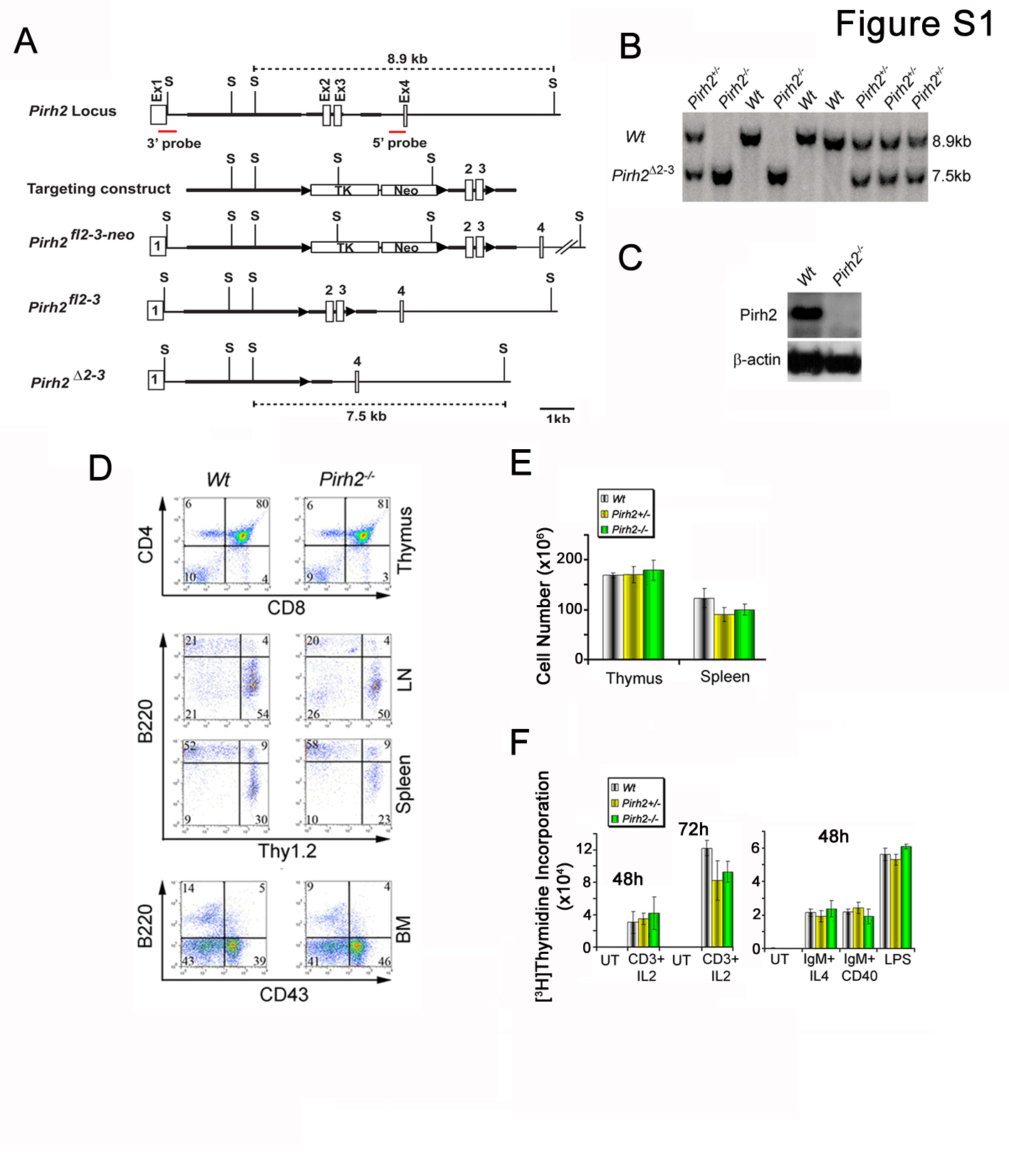

Supplement: Figure S1 — Pirh2−/− mice display normal immune cell development and proliferation of T and B-cells. (A) A schematic representation of the Pirh2 locus, the targeting construct, and the mutant Pirh2 alleles (Pirh2fl2-3-neo, Pirh2fl2-3 and Pirh2Δ2-3). Exons are indicated as boxes, loxP sites as triangles and positions of 5′ and 3′ probes used for Southern blot are indicated. S, Sst1 site. Expected sizes of Sst1 DNA fragments are indicated. (B) Southern blot analysis of Sst1 digested tail genomic DNA of a litter from Pirh2+/− intercrosses. 5′ probe shown in (A) was used. (C) Western blot analysis of cell extracts from Wt and Pirh2−/− splenocytes showing loss of Pirh2 expression in mutant cells. (D) A representative FACS analysis of thymus, spleen, LN and BM cells from 8 week-old Wt and Pirh2−/− mice. Percentages of populations are indicated. (E) Thymocyte and splenocyte numbers from 6 to 10 week-old Wt (n = 6), Pirh2 +/− (n = 5) and Pirh2−/− (n = 9) mice. No significant differences were observed between Wt and mutant mice. (F, left panel) T-cells from Wt, Pirh2 +/− and Pirh2−/− 6 to 10 week-old mice were activated using anti-CD3 and IL2 and their level of proliferation determined using [3H] Thymidine incorporation assay. Data for 48 h and 72 h time points are shown and are representative of 5 independent experiments. (F, right panel) B-cells from Wt, Pirh2 +/− and Pirh2−/− mice were activated with anti-IgM+IL4, anti-IgM+CD40, or with LPS and their proliferation determined using [3H] Thymidine incorporation assay. Data for 48 h time point are shown. This result is representative of 5 independent experiments. UT = untreated. BM: bone marrow. (TIF) [file pgen.1002360.s001.tif]

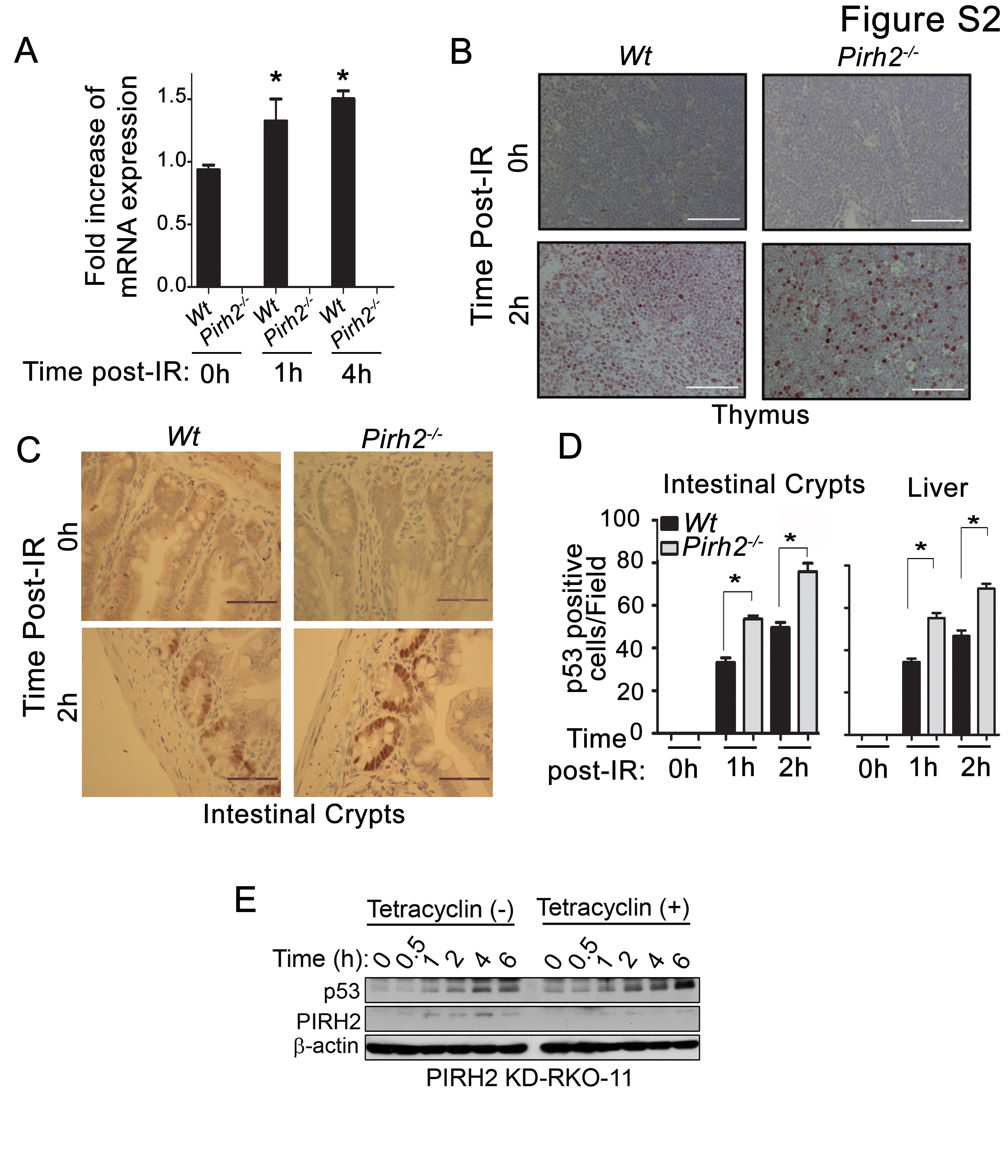

Supplement: Figure S2 — Pirh2 deficiency leads to higher accumulation of p53 in response to irradiation. (A) Splenocytes from Wt and Pirh2−/− mice were IR treated (6 Gγ) and their RNA extracted at time 0, 1 and 4 h post-IR. Quantitative RT-PCR analysis was performed to assess Pirh2 expression and was normalized to actin mRNA. Fold changes of Pirh2 mRNA expression in irradiated Wt splenocytes compared to their untreated controls (time 0 h) is shown. Student's t test was used for statistical analysis. *P<0.05 compared to time 0 h. Wt (n = 4) and Pirh2−/− (n = 5). Error bars represent SD. (B, C) 6 to 8 week-old Wt and Pirh2−/− mice either untreated or 2 h post whole-body irradiation (6 Gγ) were sacrificed and IHC was performed to assess the level of p53 in thymus (B) and intestinal crypts (C). Bar = 50 µm. (D) p53 positive cells in intestinal crypts (left) and liver (right) of untreated (n = 3) and irradiated (n = 3) Wt and Pirh2−/− mice were counted from 10 different fields for each time point. Student's t test was used for statistical analysis. * P<0.0005. Error bars represent SD. (E) A representative Western blot of three independent experiments showing the expression of p53 following tetracyclin induced PIRH2 knockdown in the human RKO cells. (TIF) [file pgen.1002360.s002.tif]

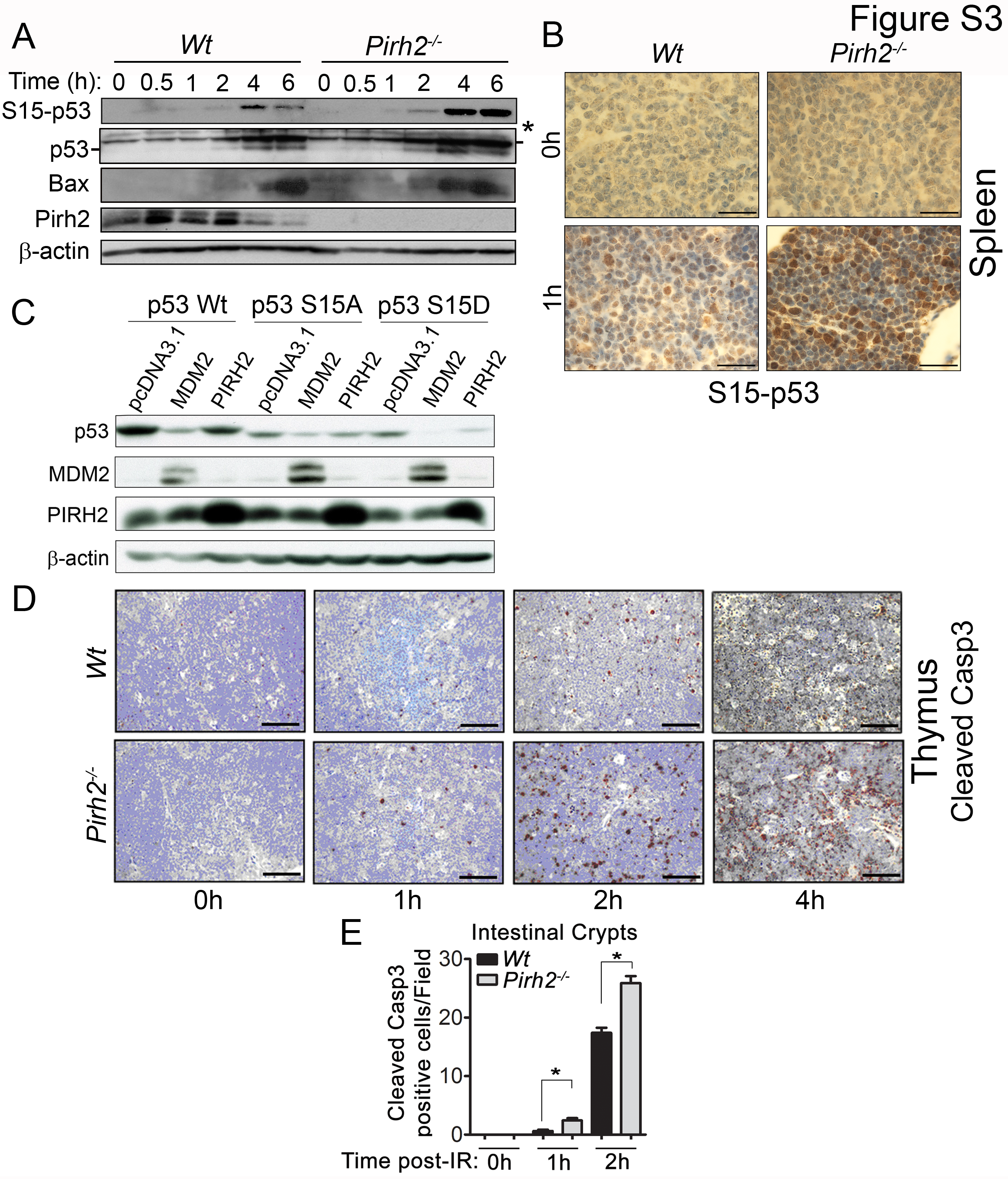

Supplement: Figure S3 — Absence of Pirh2 leads to higher levels of Serine 15 phosphorylated p53 and increased apoptosis in response to irradiation. (A) Time course immunoblot analysis of the expression level of Serine 15 phosphorylated p53 (S15-p53), total p53, Bax and Pirh2 in response to irradiation (6 Gγ) of Wt and Pirh2−/− splenocytes. *: non specific. (B) 6 to 8 week-old Wt and Pirh2−/− mice either untreated or 1 h post whole-body irradiation (6 Gγ) were sacrificed and IHC was performed to assess the level of S15-p53 in their spleen. Bar = 50 µm. (C) H1299 cells were cotransfected with either pcDNA3.1, pcDNA3.1-Mdm2 or pcDNA3.1-PIRH2 and a p53 expression vector (Wt, S15A, S15D). Lysates prepared 40 h post-transfection were examined by Western blotting using the indicated antibodies. (D) 6 to 8 week-old Wt (n = 3) and Pirh2−/− mice (n = 3) were subjected to whole-body irradiation (6 Gγ) and the levels of apoptosis in thymus at different time points post-IR were examined using active caspase 3 (casp3) and IHC. Bar = 100 µm. (E) Active casp3 positive cells in intestinal crypts of untreated (0 h) and irradiated Wt (n = 3) and Pirh2−/− (n = 3) mice were counted from 10 different fields for each time point. Student's t test was used for statistical analysis. * P<0.005. Error bars represent SD. (TIF) [file pgen.1002360.s003.tif]

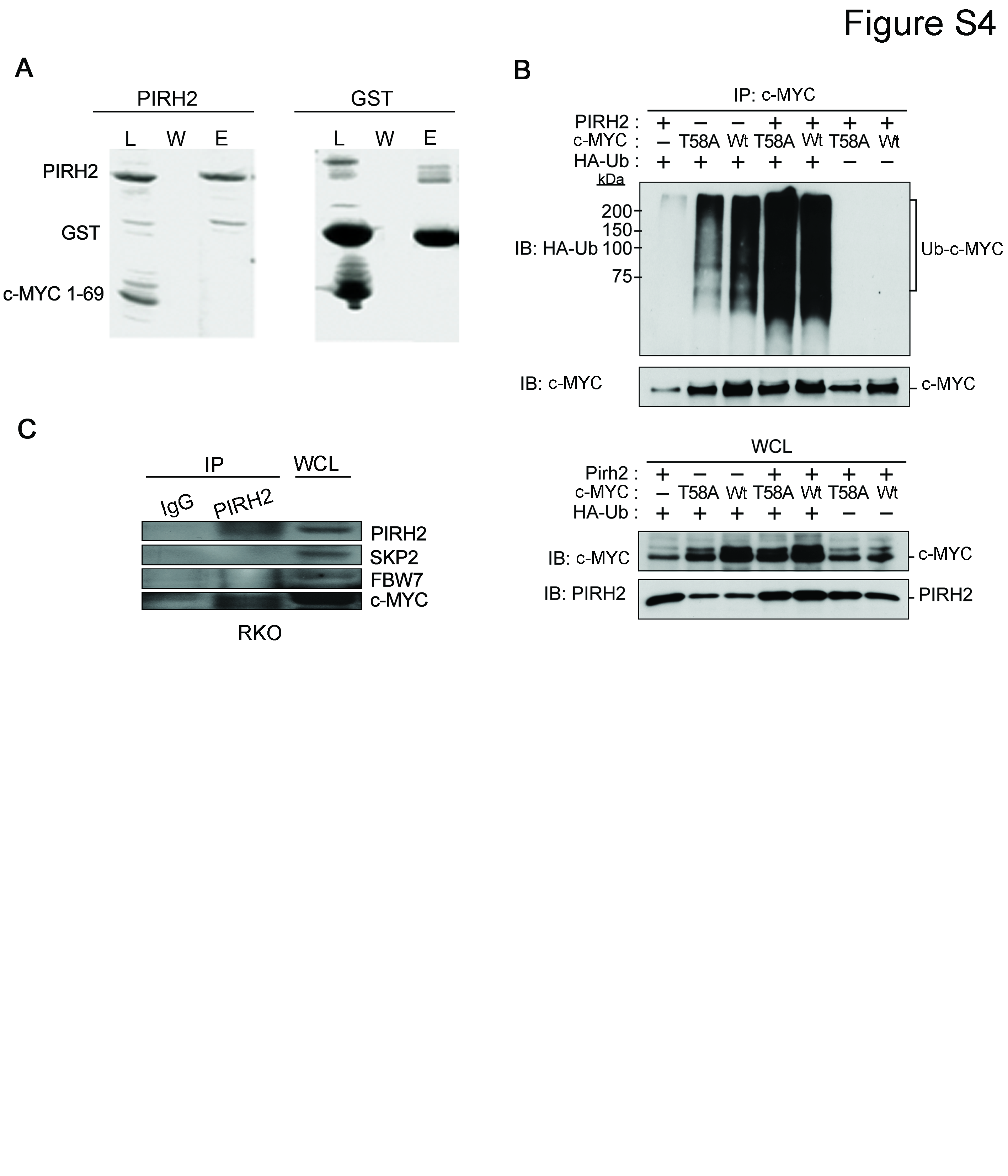

Supplement: Figure S4 — PIRH2 does not interact with SKP2 or FBW7 and ubiquitylates c-MYC independently of its phosphorylation at T58. (A) PIRH2 does not interact with c-MYCbox I (MBI). GST pull-down assays of GST-PIRH2 fusion protein with His-c-MYC boxI (1–69 aa) protein. Labeled lanes reflect loaded material (L), column flow-through after wash (W) and eluate (E). (B) Intracellular ubiquitylation assay. HEK293T cells were transfected with expression plasmids encoding Wt human PIRH2, HA-tagged ubiquitin (HA-Ub), c-MYC Wt or c-MYC T58A as indicated. IP using anti-c-MYC antibody were subjected to IB analysis with anti-c-MYC antibody (left panel). 3% of the input for IP was subjected to IB analysis with anti-cMYC and anti-PIRH2 (right panel). WCL: whole cell lysate. (C) Representative IP/Western blot data for three independent experiments showing that IP of PIRH2 from human RKO cells pulls down c-MYC but not SKP2 or FBW7. IP: immunoprecipitation. WCL: whole cell lysate. (TIF) [file pgen.1002360.s004.tif]

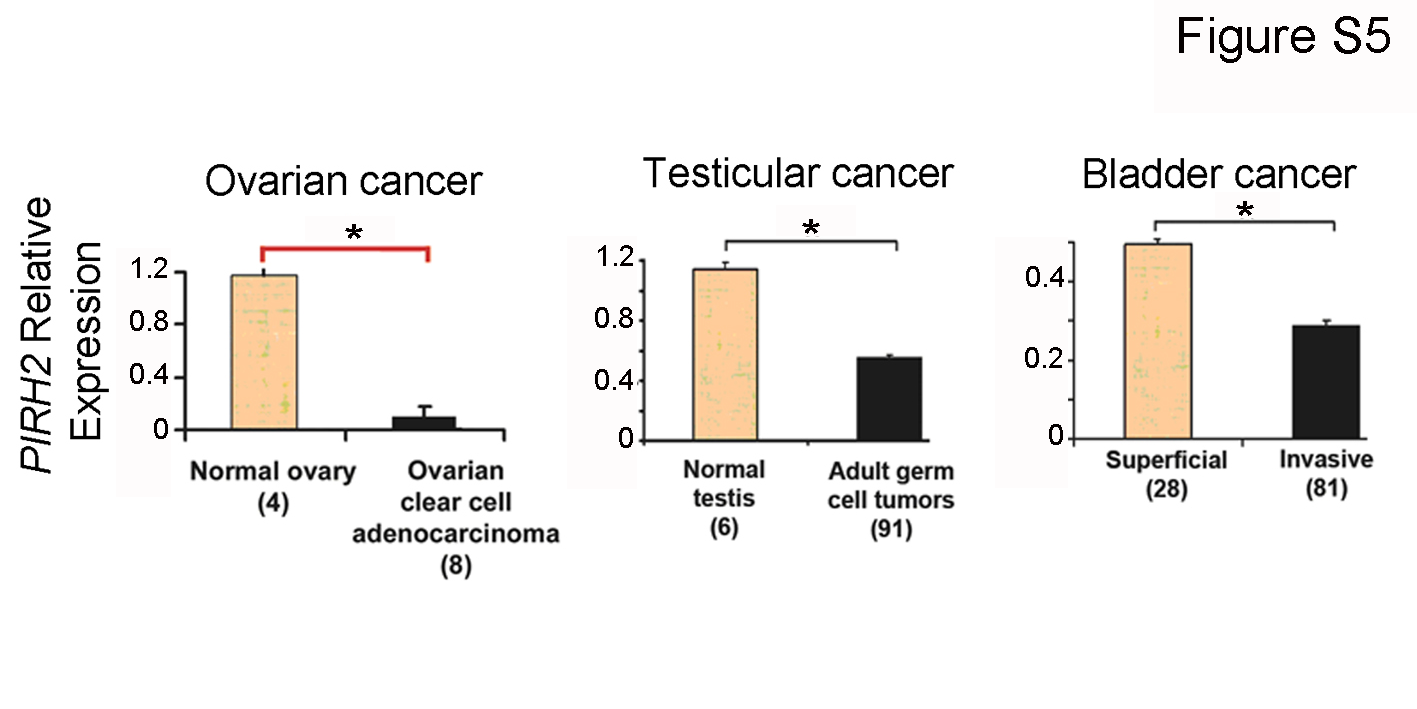

Supplement: Figure S5 — Reduced levels of PIRH2 mRNA in human cancers. (Left panel) Data [33] provided by Oncomine Research Edition v.3.6 [32] show significant downregulation of PIRH2 mRNA in ovarian clear cell adenocarcinoma compared to normal ovary (* P = 5.1×10^−6^; t-test). (E, Middle panel) Data [34] provided by Oncomine Research Edition v.3.6 show significant downregulation of PIRH2 mRNA in adult germ cell tumors compared to normal testis (* P = 1.7 10−18; t-test). (E, Right panel) Data [35] provided by Oncomine Research Edition v.3.6 show significantly downregulated PIRH2 mRNA level in invasive compared to superficial bladder cancer (* P = 4.5 10−5; t-test). (TIF) [file pgen.1002360.s005.tif]

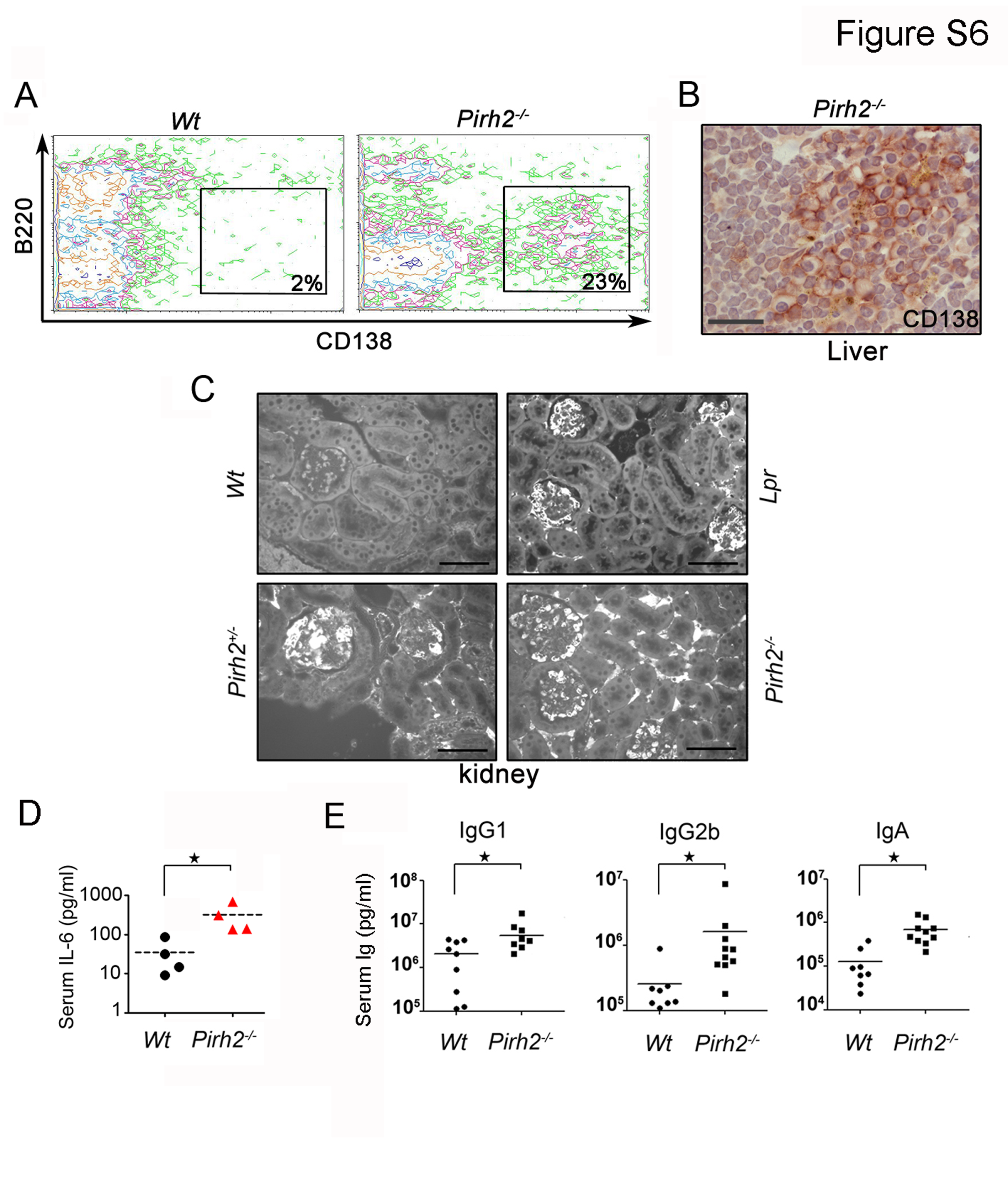

Supplement: Figure S6 — Accumulation of CD138+B220− cells, glomerular immunoglobulin deposition and elevated serum immunoglobulins in Pirh2 mutant mice. (A) Cells from spleen of 11 month-old Wt and Pirh2−/− littermates were stained with anti-CD138 (a marker for normal and malignant plasma cells), anti-B220 (a marker for B-cells) and FACS analysis was performed. The percentage of CD138+B220− cells is indicated. (B) Liver sections from a 10 month-old Pirh2−/− mouse were stained with anti-CD138. A sheet of CD138+ cells infiltrating the liver is shown. Bar = 35 µm. (C) Glomerular Immunoglobulin deposition in 10 to 12 month-old Pirh2−/− and Pirh2+/− mice. Immunoglobulin deposits in kidneys from the autoimmune Lpr mice are show as positive controls. (D) Elevated level of IL-6 in the serum of 10 month-old Pirh2−/− mice compared to Wt littermates. Student's t test was used for statistical analysis. *P<0.005. Error bars represent SD. (E) ELISA analysis of the level of IgG1, IgG2b and IgA serum Ig in 10 to 12 month-old Pirh2−/− and WT mice. * P<0.05. Bar = 50 µm. (TIF) [file pgen.1002360.s006.tif]

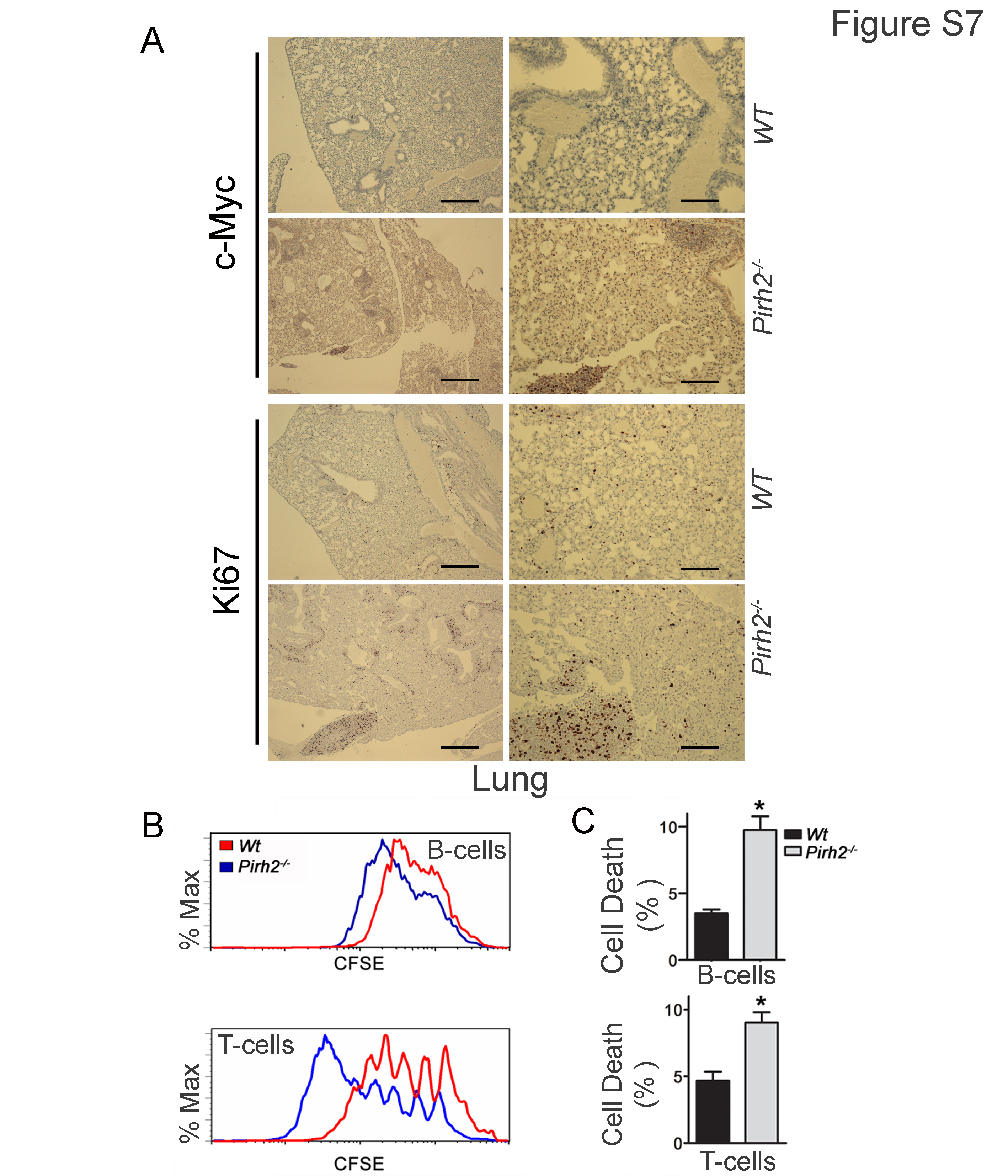

Supplement: Figure S7 — Plasma cell infiltration to non lymphoid organs of Pirh2−/− mice. IHC of the lung of a Pirh2−/− mouse showing structurally normal lung with perivascular plasma cell infiltrates. The infiltrates stain positive for c-Myc and Ki67. Lung section of Wt littermates are shown as controls. Left panels: bars = 500 µm. Right panels: bars = 35 µm. (B) FACS analysis of CFSE dilution profiles. LPS induced proliferation of CFSE-labeled B-cells (top panel) and Anti-CD3 induced proliferation of CFSE-labeled T-cells (lower panel) were examined 72 h post activation. Data are representative of four independent experiments. (C) Cell death of cells described in panel B was determined using AnnexinV/PI staining 72 h post-activation. Student's t test was used for statistical analysis. *: P<0.005. Error bars represent SD. (TIF) [file pgen.1002360.s007.tif]

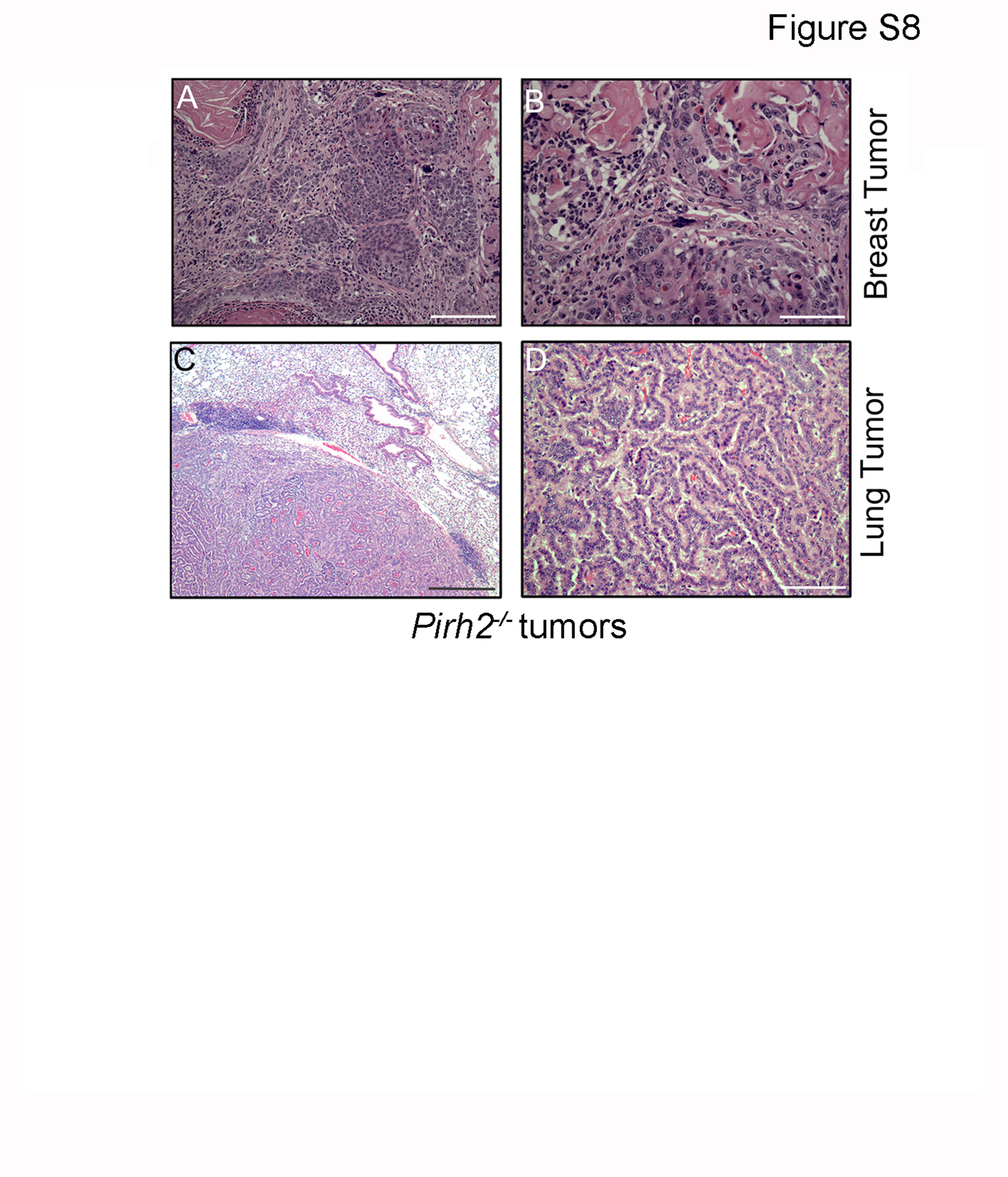

Supplement: Figure S8 — Tumorigenesis of Pirh2 mutant mice. (A, B) H&E staining of adenosquamous cell mammary carcinoma from a Pirh2−/− mouse. This tumor is composed of islets of malignant epithelial cells, with invasion to surrounding connective tissue; keratin pearls are evident within the tumor (panel A: upper and lower left corners). Higher magnification shows the glandular (panel B: lower right) and squamous cellular phenotypes (panel B: upper left and right). (A, Bar = 500 µm; B, Bar = 100 µm). (C, D) H&E staining of a Pirh2−/− lung neoplasm showing a solitary nodular mass with a well-differentiated adenomatous pattern. This lesion has well-defined borders, is highly vascularized and is surrounded by normal lung with occasional mononuclear inflammation foci. (C, Bar = 500 µm; D, Bar = 100 µm). (TIF) [file pgen.1002360.s008.tif]
