# Supplementary material for: Role of Pirh2 in Mediating the Regulation of p53 and c-Myc
Source: PLoS Genet. 2011 Nov 17;7(11):e1002360. doi: 10.1371/journal.pgen.1002360 (PMC3219591; doi:10.1371/journal.pgen.1002360)
Supplement: Table S1 — Plasma Cell Hyperplasia and Tumor Development in Pirh2 Mutant Mice. (PDF) [file pgen.1002360.s009.pdf]

Table S1. Plasma Cell Hyperplasia and Tumor Development in *Pirh2* Mutant Mice

Mouse ID	Genotype	Plasma cell hyperplasia	Tumor Type	Age (months)
1p2	*Pirh2*$^{+/-}$	Yes	Testis tumor	17
8p3	*Pirh2*$^{+/-}$	Yes	Testis tumor	6
136p9	*Pirh2*$^{+/-}$	Yes	Breast tumor	17
153p3	*Pirh2*$^{+/-}$	Yes	Breast tumor	14
86p2	*Pirh2*$^{+/-}$	Yes	Liver tumor	14
155p4	*Pirh2*$^{+/-}$	Yes	Liver tumor	14
251p3	*Pirh2*$^{+/-}$	Yes	Sarcoma	5
153p1	*Pirh2*$^{+/-}$	Yes	Lung tumor	13
82p2	*Pirh2*$^{-/-}$	Yes	Testis tumor	1
153p2	*Pirh2*$^{-/-}$	Yes	Breast squamous cell carcinoma	13
343p1	*Pirh2*$^{-/-}$	Yes	Breast tumor	6
298p2	*Pirh2*$^{-/-}$	Yes	Lung tumor	15
346p2	*Pirh2*$^{-/-}$	Yes	Lung tumor	12
370p1	*Pirh2*$^{-/-}$	Yes	Lung tumor	13
370p2	*Pirh2*$^{-/-}$	Yes	Lung tumor	14
360p1	*Pirh2*$^{-/-}$	Yes	Lung tumor	13
362p2	*Pirh2*$^{-/-}$	Yes	Liver Tumor	14
320p7	*Pirh2*$^{-/-}$	Yes	Liver Tumor	13
362p1	*Pirh2*$^{-/-}$	Yes	Uterus Tumor	12
367p5	*Pirh2*$^{-/-}$	Yes	Uterus Tumor	13
358p2	*Pirh2*$^{-/-}$	Yes	Sarcoma	14
259p5	*Pirh2*$^{-/-}$	Yes	Sarcoma	12
