# Supplementary material for: Role of Pirh2 in Mediating the Regulation of p53 and c-Myc
Source: PLoS Genet. 2011 Nov 17;7(11):e1002360. doi: 10.1371/journal.pgen.1002360 (PMC3219591; doi:10.1371/journal.pgen.1002360)
Supplement: Table S2 — Tumor types in Pirh2−/−p53−/− Mutant Mice. (PDF) [file pgen.1002360.s010.pdf]

Table S2. Tumor types in *Pirh2*$^{-/-}$*p53*$^{-/-}$ Mutant Mice

Mouse ID	Tumor Type	Age (months)
232p1	Sarcoma	5
232p6	Adenocarcinoma	3
242p4	Thymoma	5
270p3	Angiosarcoma and thymoma	5
270p6	Thymoma	4
277p1	Angiosarcoma	3
278p4	Thymoma	3
287p4	Angiosarcoma, adenocarcinoma and sarcoma	4
297p1	Adenocarcinoma	3
299p6	T lymphoma	4
306p2	T lymphoma	4
331p4	Adenocarcinoma	3
371p6	Thymoma and Angiosarcoma	4
376p1	Angiosarcoma	5
387p4	Angiosarcoma	3
